# Uncovering stem cell differentiation factors for salivary gland regeneration by quantitative analysis of differential proteomes

**DOI:** 10.1371/journal.pone.0169677

**Published:** 2017-02-03

**Authors:** Yun-Jong Park, Jin Koh, Jin Teak Kwon, Yong-Seok Park, Lijun Yang, Seunghee Cha

**Affiliations:** 1 Department of Oral and Maxillofacial Diagnostic Sciences, University of Florida College of Dentistry, Gainesville, FL, United States of America; 2 Interdisciplinary Center for Biotechnology Research, University of Florida, Gainesville, FL, United States of America; 3 Department of Oral Anatomy, Seoul National University School of Dentistry, Seoul, Korea; 4 Department of Pathology, Immunology and Laboratory Medicine, University of Florida College of Medicine, Gainesville, United States of America; University of Texas at Austin Dell Medical School, UNITED STATES

## Abstract

Severe xerostomia (dry mouth) compromises the quality of life in patients with Sjögren’s syndrome or radiation therapy for head and neck cancer. A clinical management of xerostomia is often unsatisfactory as most interventions are palliative with limited efficacy. Following up our previous study demonstrating that mouse BM-MSCs are capable of differentiating into salivary epithelial cells in a co-culture system, we further explored the molecular basis that governs the MSC reprogramming by utilizing high-throughput iTRAQ-2D-LC-MS/MS-based proteomics. Our data revealed the novel induction of pancreas-specific transcription factor 1a (PTF1α), muscle, intestine and stomach expression-1 (MIST-1), and achaete-scute complex homolog 3 (ASCL3) in 7 day co-cultured MSCs but not in control MSCs. More importantly, a common notion of pancreatic-specific expression of PTF1 α was challenged for the first time by our verification of PTF1 α expression in the mouse salivary glands. Furthermore, a molecular network simulation of our selected putative MSC reprogramming factors demonstrated evidence for their perspective roles in salivary gland development. In conclusion, quantitative proteomics with extensive data analyses narrowed down a set of MSC reprograming factors potentially contributing to salivary gland regeneration. Identification of their differential/synergistic impact on MSC conversion warrants further investigation.

## Introduction

Salivary glands (SGs) are irreversibly damaged by radiation therapy in patients with head and neck cancer or by autoreactive immune cells in Sjögren's syndrome (SjS). As a result of glandular damage, patients develop greatly diminished saliva production and feeling of dry mouth (xerostomia). The complications of dry mouth range from difficulty in speaking, swallowing, and eating, frequent fungal infections, rampant dental caries, and periodontal disease, all of which can significantly decrease the quality of life in patients [1]. At present, there is no curative therapy for these patients. Palliative treatments such as artificial saliva are limited in their effectiveness [2]. To restore normal saliva production, SG transplantation is theoretically plausible. However, organ transplantation is hampered by fundamental difficulties including the limited number of organ donors and long-lasting complications of transplantation. To circumvent the challenges, manipulation of adult stem cells has received great attention for opening new possibilities for a therapeutic intervention in patients with severe glandular damage and subsequent xerostomia.

Bone marrow (BM) includes a subpopulation of undifferentiated cells called mesenchymal stem cells (MSCs) [3, 4], which have become an important tool for cell-based therapies and tissue engineering [5, 6]. Few studies have explored MSCs for the differentiation of SG epithelial cells (SEC), which would be critical in autologous transplantation and therapeutic interventions for SjS. MSCs lessen immunoreactivity as they express the human leukocyte antigen (HLA)-G, which is a non-classical HLA class I molecule that mediates the suppressive effect of MSCs through the induction and proliferation of regulatory T cells [7]. In addition, HLA compatibility between a MSC donor and a recipient is not a major concern due to the lack of HLA-DR surface expression [8], which will alleviate any potential issues with the shortage or selectivity of donors.

Our previous published study with 2-dimensional gel electrophoresis (2-DE) proteomics on mouse BM-MSCs clearly provided a list of differentially expressed regulatory proteins and their temporal expression profiles during their differentiation into SEC in co-culture[9]. Based on the results in the study, we hypothesized that induction or suppression of key salivary gland transcription factor(TF) expression in MSCs is pivotal for MSC differentiation *in vitro* and potentially *in vivo*. Therefore, it is presumed that manipulation of these factors should allow reprogramming of these cells into the cells of interest. Although examples of TF overexpression or ablation that resulted in cell fate changes are not rare in the field of pancreatic and liver regeneration, key regulators involved in cell fate and lineage determination of MSC conversion into SECs for SG regeneration is almost non-existent.

Recently, quantitative proteomics approaches have gained popularity for elucidating underlying molecular mechanisms of stem cell differentiation [10]. With recent advances in state-of-the-art mass spectrometry (MS) techniques, studies have demonstrated that MS-based quantitative proteomics provide a powerful to identifying differentially expressed proteins in stem cell reprogramming [11–13]. One of the popular proteomics approaches includes “isobaric tag for relative and absolute quantitation” (iTRAQ). iTRAQ has been previously utilized to comprehensively analyze critical cellular processes involved in linage-specific stem cell differentiation because it provides high dynamic range of isoelectric point, identifies high and low abundant proteins, and quantitates thousands of peptides simultaneously in one analysis[14, 15].

The purpose of our study was to identify key regulatory factors for MSC conversion into SECs and identify molecular pathways involved. Therefore, we applied an iTRAQ-based proteomics analysis to investigate differentially expressed proteins during the co-culture of BM-MSCs with primary salivary gland cells (pSGCs) for 1, 3, 5, and 7 days and to map the temporal expression profiles of salivary TFs (STFs) during co-culture. Our study is the first to quantitatively analyze protein expression patterns in differentiating MSCs and to simulate potential protein networks of critical regulators for MSC reprogramming.

## Materials and methods

### Animals

C57BL/6J male mice (4–6 weeks of age) were maintained under specific pathogen-free conditions within the Animal Care Services at the University of Florida. Total of 36 mice were utilized for iTRAQ proteomics (n = 15 for three biological replicates) and co-culture for western blotting (n = 21). Both breeding and use of these animals were approved by the University of Florida Institutional Animal Care and Use Committee. The mice were euthanized according to the American Veterinary Medical Associations’ guidelines, which recommend cervical dislocation or decapitation following deep CO2 or isoflurane anesthesia.

### Mouse Bone Marrow-Derived Mesenchymal Stem Cell (mBM-MSC) culture

Mouse MSCs (mMSCs) were purchased from Life Technologies, Inc. The manufacturer isolated mMSCs from BM of C57BL/B6 mice at ≤ 8 weeks after gestation. In addition, the manufacturer reported a purity of > 95% cells positive for cell surface marker expression indicative of mMSCs (i.e. CD29^+^, CD44^+^, CD34^+^, Sca1^+^), and tested their ability to differentiate into osteocytes, adipocytes, and chondrocytes *in vitro*. For all experiments, mMSCs were cultured in our laboratory containing with 15 ml of DMEM/F12 with 10% MSC-qualified fetal bovine serum (FBS) and 5 μg/ml gentamycin and incubated in 5% CO_2_ at 37°C. mMSCs were passaged every 3–4 days when cells reached 80–90% confluence.

### Primary mouse Salivary Gland Cell (pSGC) purification and culture

mSGCs were carefully prepared to avoid contamination of other types of cells following a published protocol [16]. In short, submandibular glands tissue from 4–6 week old male C57BL/6 mice was finely cut and digested twice in Hanks’ balanced salt solution (HBSS) containing 1% (w/v) bovine serum albumin (BSA), collagenase II (0.25 mg/ml) (Life Technologies, Inc) and CaCl_2_ (6.25 mM) at 37°C for 40 minutes in a water-bath. The cell suspension was filtered with a 100 μm steel mesh and plated on non-coated 60 mm petri dish at a density of 1.2 × 10^6^ cells per plate. The petri dish was manually rotated to concentrate and harvest epithelial cells that moved towards the center of the dish. The collected pSGCs were cultured for 12 hours in the serum-free Hepato-STIM medium (BD Biocoat^TM^) with 500 U/ml penicillin/streptomycin prior to co-culturing with mMSCs. A pair of the submandibular glands yielded approximately 3.0×10^6^ to 3.5×10^6^ cells.

### Co-culture of mMSCs and pSGC

All co-culture experiments were conducted in 6- or 24-well plates containing a 0.4 μm pore size polycarbonate membrane-based transwell insert (Millipore Millicell cell culture inserts, EMD Millipore). mMSCs (1.0×10^4^ cells/cm^2^) were seeded on the collagen-coated lower chamber of the cell culture plate and incubated in Hepato-STIM media without serum for 12 hours prior to co-culture experiments. Once mMSCs were well attached to the bottom of the plate, isolated pSGCs (6×10^4^ cells/cm^2^) were seeded onto the membrane of the upper transwell insert. Cells in the co-culture system were maintained at 37°C and 5% CO_2_ without replacing the media and harvested at 1, 3, 5, or 7 days. Control mMSCs were cultured for each time point without pSGCs.

### Protein extraction and quantification

Three biological replicates of control mMSCs and co-cultured MSCs were prepared for proteomics experiments. Protein lysates were extracted and quantified following a previous method [17]. Protein assays were performed to quantify purified proteins by the Pierce™ BCA Protein Assay Kit (Thermo Fisher Scientific) with the SoftMax Pro Software v5.3 under the SpectraMax M5 (Molecular Devices, LLC). For each sample, 100 μg protein was dissolved in a dissolution buffer (AB Scienx, Inc.).

### Protein digestion, iTRAQ labeling, strong cation exchange, and LC-MS/MS

The samples were reduced, alkylated, trypsin-digested, and labeled following the manufacturer’s instructions for the iTRAQ Reagents 8-plex kit (AB Sciex, Inc.). Control mMSCs and each co-cultured mMSCs from three biological replicates were labeled with iTRAQ tags. The iTRAQ-labeled peptide mixtures were desalted with C18-solid phase extraction (The Next Group, Inc.) and fractionated with a strong cation exchange (SCX) chromatography using a polysulfoethyl A column (2.1 × 100 mm, 5 μm, 300 Å; PolyLC, Inc.) including solvent A (25% (v/v) acetonitrile, 10 mM ammonium formate, and 0.1% (v/v) formic acid (pH 2.8). Peptides were eluted with a flow rate of 200 μl/min at a linear gradient of 0–20% solvent B (25% (v/v) acetonitrile and 500 mM ammonium formate (pH 6.8) over 50 min followed by ramping up to 100% solvent B in 5 min. Sixteen fractions were collected by monitoring the absorbance at 280nm and lyophilized. A quadrupole time-of-flight (LTQ Orbitrap XL) MS system (Thermo Fisher Scientific) was applied as described previously [18]. Each fraction was loaded onto an Agilent Zorbax 300SB-C18 trap column (0.3 mm id × 5 mm length, 5 μm particle size) with a flow rate of 5 μl/min for 10 min. Reversed-phase C_18_ chromatographic separation of peptides was carried out on a pre-packed BetaBasic C_18_ PicoFrit column (75 μm id × 10 cm length, New Objective, Inc.) at 300 nl/min using the following gradient: 5% B for 1 min as an equilibration status; 60% B for 99 min as a gradient; 90% B for 5 min as a washing status; 5% B for 10 min as an equilibration status (solvent A: 0.1% formic acid in 97% water, 3% ACN; solvent B: 0.1% formic acid in 97% ACN, 3% water).

### iTRAQ LC-MS/MS data analysis

The MS/MS data were processed by a comprehensive search, considering amino acid substitution against the UniProt *Mus musculus* FASTA database (87,273 entries, http://www.uniprot.org) using ProteoIQ v2.7 (PREMIER Biosoft), ProteinPilot v4.5 (AB Sciex) with the Paragon^TM^ algorithm [19], Proteome Discoverer v1.4 (Thermo Fisher Scientific) with the SEQUEST algorithm [20], and Mascot v2.3 (Matrix Science). The following parameters were used for all searches: peptide tolerance at 10 ppm, tandem MS tolerance at ± 0.01 Da, peptide charges of 2+ to 5+, trypsin as the enzyme allowing one missed cleavage, iTRAQ label and methyl methanethiosulfonate. After searching MS/MS spectra against this database, the results of matched peptide number and their expression pattern were combined into each group from all individual batches. Systematic bias was corrected using intensity normalization of protein quantification across samples, and peptide and protein were filtered using ProteoIQ2.7 (PREMIER Biosoft) with strict peptide and protein probabilities, 0.95 respectively. ProteoIQ filtered peptide with a minimum probability of >0.95 and a minimum protein probability of <0.95 from all search results for final identification at a 5% false discovery rate (FDR) [21]. Protein relative quantification was performed using the ratios from MS/MS spectra when the peptides were uniquely assigned to a detected protein. To be identified as being significantly differentially expressed, a protein had to be quantified with at least three peptides in experimental replicates, a p value <0.05, and a fold change >1.3 or <0.7 as determined with a Fisher’s combined probability of < 0.05. The mass spectrometry proteomics data have been deposited to the ProteomeXchange Consortium [22] via the PRIDE partner repository with the dataset identifier PXD002796 and 10.6019/PXD002796.

### Western blot analysis

Following a conventional protocol, aliquots of 20 μg of each sample were mixed with loading buffer (60 mM Tris-HCl, 25% glycerol, 2% SDS, 14.4 mM 2-ME, 0.1% bromophenol blue), separated on 4–15% gradient SDS-PAGE gels, and transferred to a PVDF membrane (Bio-Rad Inc.). Membranes were blocked for 1 h with 5% non-fat dry milk and incubated with antibodies against α-amylase, Ptf1α and Mist-1 (Santa Cruz Biotechnology, Inc.). Blots were washed and then incubated for 1 h at room temperature with horseradish peroxidase (HRP)-conjugated anti-rabbit or anti-goat antibodies. Sites of antibody binding were visualized by enhanced chemiluminescence (ECL; GE Healthcare Life Sciences) western blotting detection system, and quantified using a densitometer analysis (ImageJ; http://rsb.info.nih.gov/ij).

### Immunocytochemistry

Co-cultured or control mMSCs grown on coverslips (BD BioCoat™) were fixed with 4% paraformaldehyde for 20 minutes. After fixation, the cells were washed, permeabilized, and blocked in phosphate-buffered saline (PBS) containing 0.2% Triton X-100 and 5% FBS for 30 minutes at room temperature. Cells were then incubated with goat polyclonal anti-Ptf1α (1: 200), rabbit polyclonal anti-Mist-1 (1: 200) in PBS containing 0.1% Triton X-100 and 1% FBS at 4°C overnight. After washing with PBS, the cells were incubated at room temperature for 1 h with appropriate fluorescence-conjugated secondary antibodies (1: 200 dilution, Molecular Probes). Coverslips were mounted and nuclei stained with Vectashield mounting solution containing DAPI (Vector Laboratories Ltd). Fluorescence was observed under a 100X magnification using a Zeiss Axiovert 200M microscope equipped with a Zeiss AxioCam MRm camera and images were obtained from AxioVs40 software (Ver. 4.7.1.0, Zeiss).

### Functional annotation and protein network analysis

Functional annotation was conducted by PANTHER (http://www.pantherdb.org) and Blast2GO (http://www.blast2go.com/b2ghome) [23]. Blast2GO level 3 filtering was used to examine unique protein changes during comparison analysis. To examine if any of differentially expressed proteins detected by iTRAQ were involved in the developing SG, mRNAs known to be expressed in the developing SGs at the NIDCR website (http://sgmap.nidcr.nih.gov/sgmap/sgexp.html) were examined. For pathway analysis of six TFs and identified proteins, STRINGSv.9.1(http://string-db.org)[24] and WikiPathway (http://www.wikipathways.org/index.php/WikiPathways)[25] were utilized. These programs analyze hypothetical protein interaction clusters based on a regularly updated database, which consists of millions of individual relationships among proteins gathered from the biologic literature.

### Experimental design and statistical rationale

All data with normal distribution are presented as mean ± standard error of over three independent experiments. Significance of differences was determined by one-way ANOVA with Bonferroni *post-hoc* test using JMP 9.0.1 (SAS Institute Inc., Cary, NC, USA) to determine whether there are differences in protein expression among the control and other differentiated cells.

## Results

### Analysis of the proteome data set and Gene Ontology (GO) classification

**Fig 1:**
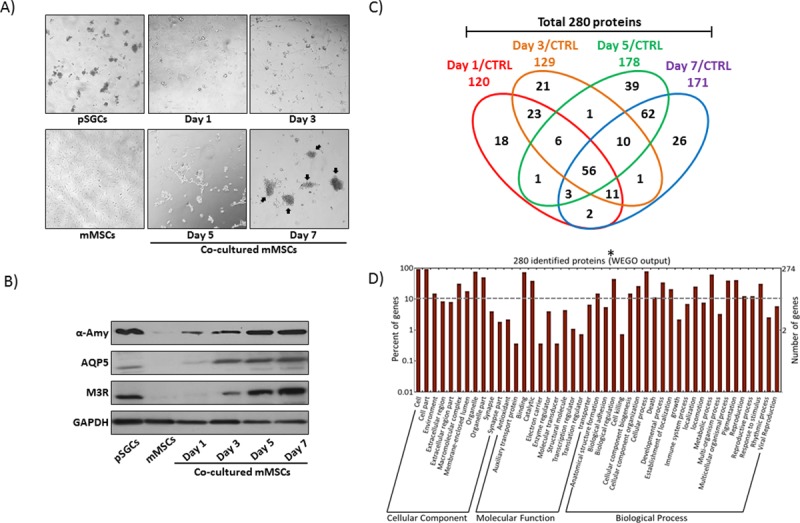
Verification of morphological changes of co-cultured MSCs and functional category of differentially expressed proteins following iTRAQ. (A) Microscopic images of C57BL/6-derived pSGCs (upper, first panel), control mMSCs (lower, first panel), and co-cultured MSGs at day 1, 3, 5 and 7 were shown at a 20X magnification. pSGCs showed a typical islet-like cell morphology while control mMSCs exhibited fibroblast-like cell appearance as predicted. Aggregated co-cultured MSCs, which morphologically resembled islet-forming pSGCs, at each culture time point were indicated by black arrowheads in the day 7 panel. Co-culture was carried out for 7 days without replacing media. (B) Total protein lysate isolated from co-cultured mMSCs was subject to western blotting. Control mMSCs were used as a negative control while pSGCs were used as a positive control. Three acinar cell maker proteins (a-amylase, a-Amy; aquaporin-5, AQP5; and muscarinic acetylcholine receptor type-3, M3R) were detected in pSGCs and co-cultured mMSCs. (C) *A Venn diagram* showed the distribution of number of differentially expressed 280 proteins, selected from statistical analyses of iTRAQ data, at each time point during co-culture. (D) A total of 280 identified proteins were assigned to 45 functional groups using Gene Ontology (GO). All identified unigenes were aggregated into three main categories: Cellular Component, Molecular Function, and Biological Process. Percentages are based on the proportion and number of genes in each set. *WEGO (Web Gene Ontology Annotation Plot).

In our previous study, we reported that BM-MSCs differentiated into SECs when co-cultured with mouse pSGCs in serum free-Hepato-STIM media without cell-to-cell contact. This was based on the fact that differentiating MSCs in co-culture expressed prototypic SEC marker proteins, such as SG-specific α-amylase (AMY1), aquaporin-5 (AQP5), and type 3 muscarinic receptor (M3R) **[9]**. Prior to performing our current iTRAQ-LC-MS/MS analysis, we reproduced our results from the previous study, demonstrating again the morphological changes of mMSCs during co-culture (Fig 1A). In our biological replicates, fibroblast-like MSC stem cells differentiated into round epithelial-like cells during 7 days of co-culture, eventually resembling isolated pSGCs. In contrast, control mMSCs without pSGC co-culture under the same culture condition sustained typical MSC morphology. SG marker protein expression was also confirmed by western blotting as indicated in Fig 1B, ensuring the result from our co-culture is reproducible. Then, we performed 8-plex iTRAQ-LC-MS/MS with three sets of biological replicates (set#1 through set #3 in Fig A in S1 File) of differentiated MSCs for protein identification and quantification at each co-culture time point (1, 3, 5, and 7 days). When we used a 95% cut-off value from three different sets, 1,844 distinct proteins were quantified as differentially expressed proteins (Raw data files were uploaded at PRIDE; http://www.ebi.ac.uk/pride/archive; Project Accession: PXD002796) and matched against the Uniprot database (Uniprot_Mouse20130625.fasta) (Fig A in S1 File). 1844 proteins were subject to volcano plot analysis with a 30% cut-off value, which categorized 280 proteins (15.19%) as differentially expressed proteins in co-cultured MSCs compared to control MSCs. The proteins were listed in Fig B in S1 File and Table A in S2 File. As shown in the *Venn Diagram* in Fig 1C, the expression of 56 proteins from the 280 protein pool (20%) was altered throughout the co-culture period.

Next, to categorize various regulatory factors based on their putative functions, Gene Ontology (GO) functional categories were assigned to 280 proteins, such as “Cellular Component”, “Molecular Function”, and “Biological Process” (http://www.geneontology.org) for GO enrichment analysis. As shown in Fig 1D, the output of WEGO tool indicates that greater enrichment was found to be in “Biological Processes”, especially in the categories of biological regulation, cellular component biogenesis and organization, developmental process, establishment of localization, metabolic process, multicellular organismal process, and response to stimulus. In order to verify specific functions of proteins that are involved in cell differentiation, we utilized categories that were used for “Biological Process” for further analysis of putative protein functions herein.

### Protein clusters and cellular function analysis of differentially expressed proteins

**Fig 2:**
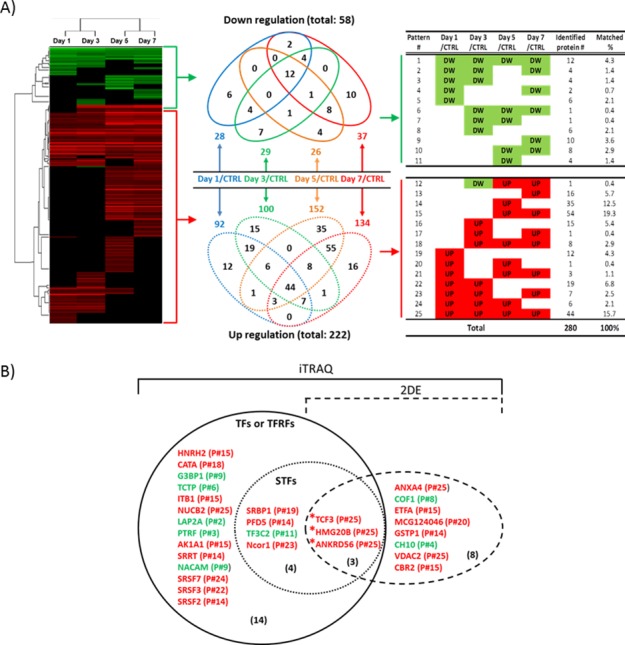
Heatmap and expression analysis of proteins for each culture time point and identification of transcription factors. (A) Of 280 proteins, proteins were categorized via heatmap analysis, based on their expression pattern at each time point, into upregulated protein expression (colored in red) and downregulated proteins (colored in green). Up and down-regulated proteins were also presented in a *Venn diagram* separately, showing unique or concurrent protein expression in differentiating MSCs at a given culture time point. These 280 differentially expressed proteins were clustered into 25 different patterns based on their temporal expression profiles (right panel; DW, down; UP, up; matched %, % of number of identified proteins out of 280). (B) TFs and TFRF were identified out of the 280 proteins. Of these, 11 factors were detected in our previous data using 2-DE. Three TFs from our two different analyses of iTRAQ and 2-DE, were regarded as STFs by the NIH/NIDCR database. All listed proteins were represented with gene abbreviation and the full names were listed in Table A in S2 File. Asterisks in red indicate differentially expressed salivary gland transcription factors that were detected both by iTRAQ and 2-DE.

Of the 280 differentially expressed proteins, we identified upregulated (red) or downregulated (green) proteins in co-cultured MSCs at each time point as indicated by the heat-map in Fig 2A. Hierarchical cluster analysis revealed a marked difference between the proteins expressed in control MSCs and those in differentiating MSCs over time. We observed that expression of 58 proteins was down-regulated (fold change <0.7) and of 222 proteins was up-regulated (fold change >1.3) relative to control mMSCs. Then proteins were categorized into *venn diagrams* based on the protein expression patterns over the course of 7 days in comparison with MSC controls. Among the up-regulated proteins, 40.7% of proteins (114 proteins from patterns #13, #15, and #25) were expressed during the later culture period, especially at day 5 and/or day 7 (Table in Fig 2A). In our previous study, we identified differentially expressed proteins in co-cultured mMSC by applying 2-DE [9]. Interestingly, 11 of those detected by 2-DE were also detected by iTRAQ as differentially expressed proteins (Fig 2B). Furthermore, the NIH/NIDCR database (http://sgmap.nidcr.nih.gov/sgmap/sgexp.html) search revealed three proteins out of 11 detected by both 2-DE and iTRAQ as STFs involved in SG development. These proteins include transcription factor E2a (TCF3), high mobility group proteins 20B (HMG20B), and ankyrin repeat domain-containing protein 56 (ANKRD56). In addition, as shown in Fig 2B, SREBP-1A-W42 of sterol regulatory element binding proteins 1 (SRBP1), prefoldin subunit 5 (PFD5), general transcription factor 3C polypeptide 2 (TF3C2), and Ncor1 protein, which were newly detected by robust iTRAQ, were also categorized as STFs by the same database. Furthermore, fourteen TFs and transcription regulatory factors (TFRFs) detected by iTRAQ and analyzed by Blast2GO were additionally selected for proteins of interest. Interestingly, most of these identified TFs and TFRFs belong to a group of up-regulated proteins (indicated in red) in the right panel shown in Fig 2A.

### Protein enrichment of differentially expressed proteins

**Fig 3:**
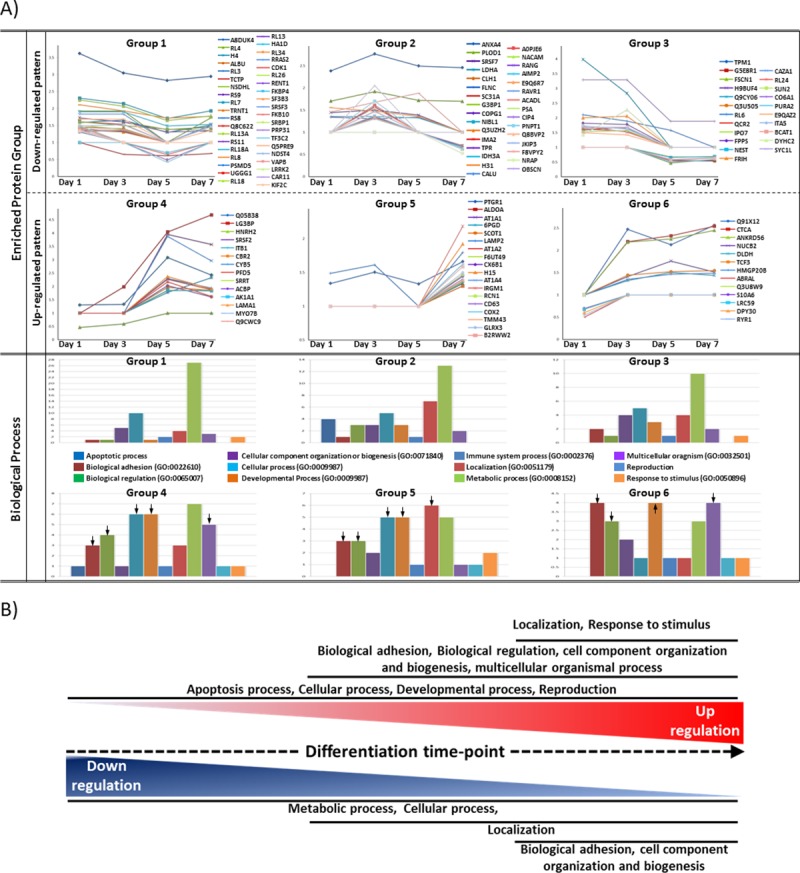
Cluster analysis of differentially expressed proteins and categorization by protein functions. (A) Differentially expressed proteins were clustered into six different categories with Groups 1–3 representing down-regulated proteins while Groups 4–6 representing upregulated proteins. In Group 1, 39 proteins decreased significantly at all four culture time points (1, 3, 5 and 7 days). In Group 2, 31 proteins were downregulated after 3 days of co-culture. In Group 3, the expression levels of 22 proteins decreased after 5 days of co-culture. In Group 4, 14 proteins showed gradual increase in expression during the 7 day course of co-culture. In Group 5, 18 proteins significantly increased in their expression on day 7. For Group 6, 13 proteins had increased expression by day 3. All categorized proteins were then evaluated for their functional annotations according to the PANTHER and BlastGO classification systems. Proteins were further enriched in a variety of cellular functions and their functionality is briefly summarized. Arrowheads indicate major cellular functions in Groups with upregulated expression patterns compared with Groups with downregulated patterns. (B) Schematic representation of potential functions of differentially expressed proteins throughout the culture period.

All analyzed proteins were further clustered into six enriched protein groups according to their temporal expression profiles (Fig 3A). “Down-regulated pattern” in Fig 3A includes Groups 1, 2, and 3, representing downregulation after day 1, 3, and 5, respectively. “Up-regulated pattern” includes Groups 4, 5, and 6, showing increased protein expression after day 3, day 5, and by day 3, respectively. Proteins that belong to each group were also listed in Table B in S2 File.

Furthermore, each cluster was evaluated by Blast2Go and PANTHER software (www.pantherdb.org) to identify key biological processes associated with those expression patterns. These analysis methods revealed that enriched protein groups over time are associated with a wide array of biological processes, which was summarized as bar graphs under “Biological process” (Fig 3B). For the proteins in Groups 1 through 3 appear to be associated with metabolic and cellular processes, cellular localization, biological adhesion and regulation, cellular component organization or biogenesis, immune system process, and multicellular organismal processes. In addition, proteins clustered into Group 4 were mainly associated with apoptosis, cellular and developmental processes, and reproduction. Group 5 proteins were mainly categorized into biological regulation, cell component organization and biogenesis, and multicellular organismal process. Lastly, most proteins of Group 6 were related to localization and response to stimulus functions (Fig 3A). Interestingly, proteins related to developmental process, biological adhesion, biological regulation, cellular process, localization, and multicellular organismal process (arrow heads) were mainly enriched in the category of “upregulated pattern”.

Our analyses of expression patterns with putative functions indicate that Group 4 includes proteins identified in the patterns #14 and #15 (HNRH2, ITB1, AK1A1, SRRT, PFD5 and SRSF2) in Fig 2A as newly identified TFs in *Venn diagrams* in Fig 2B. Moreover, proteins in the patterns #18 and #25 (CATA, NUCB2, HMGP20B, TCF3, and ANKRD56) were classified into Group 6. Taken together, 52.3% of TFs (11 TFs/ total 21 TFs in Fig 2B) belong to Groups 4 and 6, which appear to be highly associated with developmental process. In Fig 3B, biologically active processes in differencing MSCs were summarized and depicted, based on up- or down-regulated protein enrichment analyses.

### Novel protein expression

**Fig 4:**
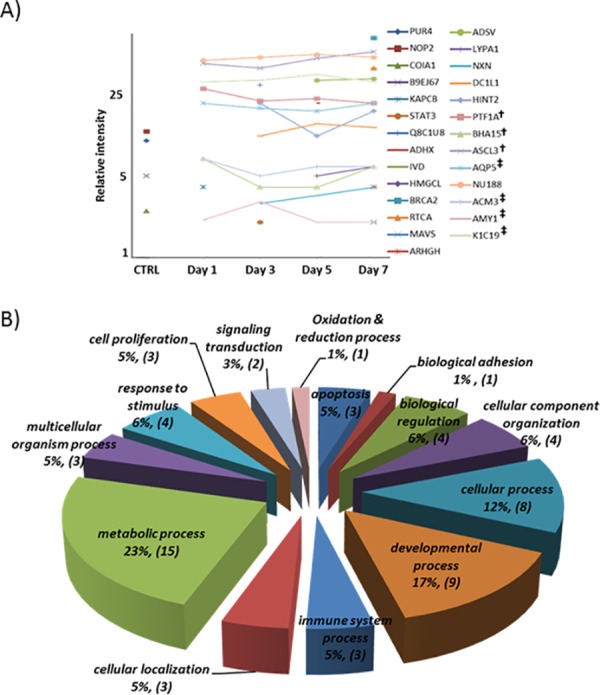
Functional categorizations of proteins detected only either in control MSCs or in co-cultured MSCs. (A) Expression intensity (arbitrary units) of 27 newly detected proteins at all time points was depicted. The four points in Ctrl indicate 4 proteins detected only in control MSCs. (^†^, newly induced SG TFs; ^‡^, SG marker proteins) (B) Twenty seven newly detected or suppressed proteins in differentiating MSCs were categorized by their predicted functions and presented as a pie chart. The pie chart shows the number of proteins that belong to each functional category as a percentage along with actual numbers in the parenthesis. PANTHER and BlastGO software were utilized for this analysis.

In addition to differentially expressed proteins described above, we identified 23 proteins that were newly induced only in co-cultured MSCs at least one time point whereas 4 proteins were detected only in control MSCs during co-culture (Table 1). These 27 total proteins were not evaluated or analyzed as part of 280 differentially expressed proteins because they were only expressed either in control MSCs or in differentiated MSCs during co-culture but not in both. No fold change of protein expression relative to that in the control could be calculated as a result. Therefore, they were classified as “novel proteins”. Most interestingly, in the list of 27 novel categorized proteins, SG acinar cell-specific markers of AMY, M3R and AQP5 were detected as newly induced proteins at all time points and SG ductal cell marker of CK19 was also detected during co-culture (marked as **‡)**, as consistent with our previous study [9]. These proteins were not detected in control MSCs (Table 1 and Fig 4A). Moreover, TFs such as PTF1α, MIST-1 (BHA15), and ASCL3 (marked as †) were also identified to be newly induced during co-culture (Table 1).

**Table 1:** List of newly induced or suppressed proteins and their biological process following co-culture.

No	GenBank ID	Gene Name	Gene Abbrev.	GO Biological process		Co-culture (Day)			
					Ctrl	1	3	5	7
1	sp|Q5SUR0|	Phosphoribosylformylglycinamidine synthase	PUR4	MP,	**P**	**-**	**-**	**-**	**-**
2	sp|Q922K7|	Putative ribosomal RNA methyltransferase	NOP2	CP	**P**	**-**	**-**	**-**	**-**
3	sp|P39061-1|	Isoform 2 of Collagen alpha-1(XVIII) chain	COIA1	BA, BR, CCO, CP, DP, CL, MOP, RS	**P**	**-**	**-**	**-**	**-**
4	tr|B9EJ67|	G protein-coupled receptor 111	B9EJ67	ST,	**P**	**-**	**-**	**-**	**-**
5	sp|P68181-2|	Isoform 2 of cAMP-dependent protein kinase catalytic subunit beta	KAPCB	CP, MP, MOP,	**-**	**P**	**-**	**-**	**-**
6	sp|P42227-2|	Isoform Stat3B of Signal transducer and activator of transcription 3	STAT3	AP, BR, CP, DP, ISP, MP, RS	**-**	**-**	**P**	**-**	**-**
7	tr|Q8C1U8|	Putative uncharacterized protein (Fragment)	Q8C1U8	n/d	**-**	**-**	**P**	**-**	**-**
8	sp|P28474|	Alcohol dehydrogenase class-3	ADHX	AP, DP, MP,	**-**	**-**	**-**	**P**	-
9	sp|Q9JHI5|	Isovaleryl-CoA dehydrogenase, mitochondrial	IVD	ORP	**-**	**-**	**-**	**-**	**P**
10	sp|P38060|	Hydroxymethylglutaryl-CoA lyase, mitochondrial	HMGCL	MP,	**-**	**-**	**-**	**-**	**P**
11	sp|P97929|	Breast cancer type 2 susceptibility protein homolog	BRCA2	AP, DP,	**-**	**-**	**-**	**-**	**P**
12	sp|Q9D7H3|	RNA 3'-terminal phosphate cyclase	RTCA	MP,	**-**	**-**	**-**	**-**	**P**
13	sp|Q8VCF0|	Mitochondrial antiviral-signaling protein	MAVS	CP, ISP, RS	**-**	**-**	**-**	**-**	**P**
14	sp|Q80U35|	Rho guanine nucleotide exchange factor 17	ARHGH	ST,	**-**	**-**	**P**	**-**	**P**
15	sp|Q60604-2|	Isoform 2 of Adseverin	ADSV	CCO, CP, DP,	**-**	**-**	**-**	**P**	**P**
16	sp|P97823-2|	Isoform 2 of Acyl-protein thioesterase 1	LYPA1	CP, MP,	**-**	**-**	**-**	**P**	**P**
17	sp|P97346-2|	Isoform 2 of Nucleoredoxin	NXN	DP, ST	**-**	**-**	**P**	**P**	**P**
18	sp|Q8R1Q8|	Cytoplasmic dynein 1 light intermediate chain 1	DC1L1	CCO, DP, CL, MP,	**-**	**-**	**P**	**P**	**P**
19	sp|Q9D0S9|	Histidine triad nucleotide-binding protein 2, mitochondrial	HINT2	MP,	**-**	**-**	**P**	**P**	**P**
20	sp|Q9QX98|	Pancreas transcription factor 1 subunit alpha	PTF1A	DP,	**-**	**P**	**P**	**P**	**P**
21	sp|Q9QYC3|	Class A basic helix-loop-helix protein 15	BHA15	BR, DP, MP,	**-**	**P**	**P**	**P**	**P**
22	sp|Q9JJR7|	Achaete-scute homolog 3	ASCL3	BR, DP, MP,	**-**	**P**	**P**	**P**	**P**
23	sp|Q6ZQH8|	Nucleoporin NUP188 homolog	NU188	MP	**-**	**P**	**P**	**P**	**P**
24	sp|Q9WTY4|	Aquaporin-5	AQP5	CL,	**-**	**P**	**P**	**P**	**P**
25	sp|Q9ERZ3|	Muscarinic acetylcholine receptor M3	ACM3	CP, ISP, MOP,RS	**-**	**P**	**P**	**P**	**P**
26	sp|P00687|	Alpha-amylase 1	AMY1	MP,	**-**	**P**	**P**	**P**	**P**
27	sp|P19001|	Keratin, type I cytoskeletal 19	K1C19	CCO, CP, DP,	**-**	**P**	**P**	**P**	**P**

AP, apoptosis; BA, biological adhesion; BR, biological regulation; CCO, cellular component organization; CP, cellular process; DP, developmental process; ISP, immune system process; CL, cellular localization; MP, metabolic process; MOP, multicellular organism process; RT, response to stimulus; CP, cell proliferation; ST, signal transduction; ORP, oxidation and reduction process; P, Positive detection; -, not detected.

Relative expression intensity levels of these newly induced proteins were graphed at each time point (Fig 4A). In addition, novel proteins were further evaluated based on “Biological Process” using the Blast2GO and PANTHER program, as summarized in Fig 4B. Interestingly, we identified 5 proteins known as TFs, such as ASCL3, MIST-1(BHA15), STAT3, ASCL3, and PTF1a in developmental process. Other proteins known to be involved in developmental stage such as ADSV, DC1L, and ADHX were newly expressed at later culture time points (5 and/or 7 days). In contrast, COIA1 identified in developmental process was expressed only in the control MSCs (Fig 4A).

### Expression verification of two putative key regulators for MSC conversion into SEC

**Fig 5:**
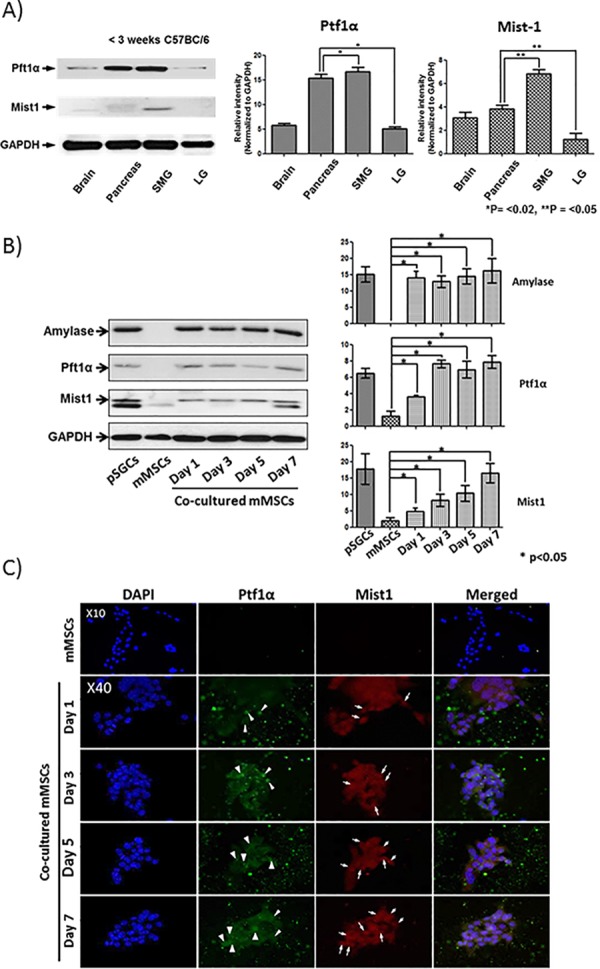
Confirmation of Ptf1a and Mist1 expression by western blotting and immunocytochemistry. (A) Total protein lysates isolated from the brain, pancreas, submandibular gland (SMG) and lacrimal gland (LG) tissues of 4 week-old C57BL/6J mice were probed by western blotting for Ptf1α and Mist-1. Isolated proteins from the brain and pancreas were used as positive protein controls for Ptf1α and Mist-1. GAPDH protein was used as a loading control. (B) Proteins lysate prepared from all co-cultured mMSCs were probed by western blotting for expression of AMY, Ptf1α, Mist-1, and GAPDH. SMX was used as a positive control and control mMSCs was used as a negative control. *p<0.05, **p<0.02 with one-way ANOVA with Bonferroni post hoc. (C) Immunocytochemistry of co-cultured mMSCs was probed for expression of Ptf1α and Mist-1. DAPI was used to stain nuclei. Arrows indicate cell-specific expression of proteins of interest during co-culture.

We hypothesized that newly expressed proteins after co-culture of MSCs with pSGCs would be critical for MSC differentiation into SEC by changing of molecular machinery. We selected Ptf1α and Mist-1 as molecules of interest as they were detected only in the differentiated MSCs during co-culture and our database search confirmed that Ptf1α and Mist-1 are TFs that are most likely involved in developmental process, as stated earlier. We further verified protein expression by western blotting utilizing exocrine tissues of the brain, pancreas, submandibular glands (SMG) and lacrimal glands (LG) (Fig 5A) from mice and evaluated for expression of Ptf1α and Mist-1. The brains and pancreas were used as a positive control for Ptf1α and Mist-1. To our surprise, Ptf1α was highly expressed in SMG, similar to the level detected in the positive control of pancreas, and in LG to a lesser degree. Similarly, Mist-1 was also significantly expressed in SMG compared with the brain and the pancreas but only slightly expressed in LG (Fig 5A). The protein expression patterns of Ptf1α and Mist-1 were further confirmed following a new set of co-culture experiments using western blot analysis and were significantly elevated as the culture progressed (Fig 5B). Furthermore, immunocytochemistry evaluation of protein expression also revealed positive signals for Ptf1α and Mist-1 in cellular cytoplasm and nucleus in a time dependent manner (arrowheads in Fig 5C), which was consistent with our iTRAQ data.

### Pathway analysis of selected proteins during transdifferentiation

**Fig 6:**
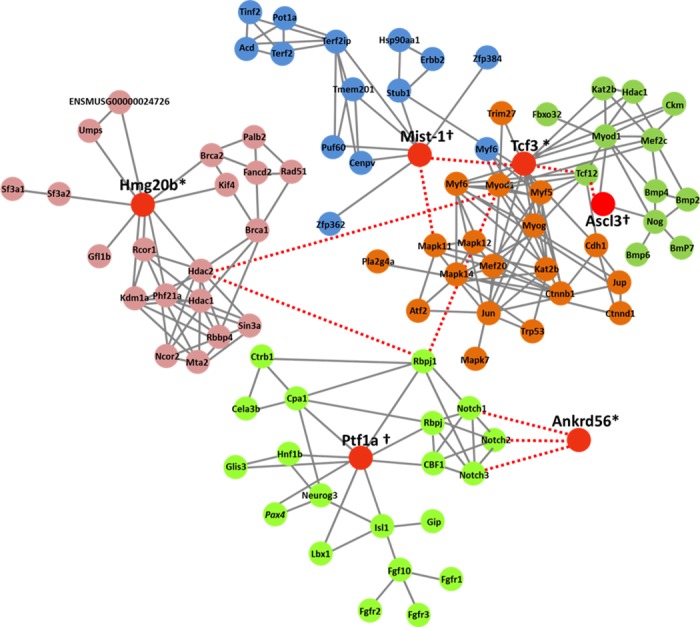
Molecular network analysis of six transcription factors of interest. Protein interaction pathways among six selected TFs were presented. Red circles indicate main transcription factors of interest (Tcf3, Mist-1, Hmg20b, Ptf1α, Ascl3, and Ankrd56) using functional analysis assessment. Green, proteins related to Ascl3; Brown, proteins directly regulated by Tcf3; Blue, proteins that were identified with a functional relationship with Mist-1(Bhlha15); Light pink, Hmg20b-related proteins; Light green, proteins associated with Pft1a. All these interactome analyses among six TFs were performed using STRING protein interaction database. Only the proteins with known and predicted interactions are shown in the schematic representation. A search for Ankrd56 did not yield its related proteins. The gray non-dashed lines between proteins represent high-confidence interaction, whereas red dashed lines between proteins represent low-confidence interactions from STRING database. *, differentially expressed proteins detected by 2-DE and iTRAQ; †, proteins induced only in co-cultured MSCs detected by iTRAQ.

Proteins playing a pivotal role in a specific biological process can be identified through the pathway analysis. We were interested in three differentially expressed TFs (Tcf3, Hmg20b and Ankrd56) identified by 2-DE [9] and iTRAQ (Fig 2B), and three additional TFs (Ptf1α, Mist-1 and Ascl3) identified as newly induced proteins by iTRAQ (Fig 4A) for further analyses of their putative functions. Our literature search indicates those 6 proteins appear to be associated with “differentiation” in general and/or exocrine glandular differentiation (Table 2). Fig 6 visualized and summarized the network of predicted associations between each TF of interest and a particular group of proteins using STRING database.

**Table 2:** List of six transcription factors and their cellular functions reported in literature.

	Gene Name	Function	References
Group6	TCF3 (Transcription Factor E2A)	Determine tissue-specific cell fate during embryogenesis, Control self-renewal and regulate the lineage, Interact with PTF1ɑ	[70–74]
	HMG20B (High Mobility Group Protein 20B)	Role in cell cycle regulation and effective cell fate	[75]
	ANKRD56 (Ankyrin Repeat Domain-containing Protein 56)	Act as a regulator for cell fate through Notch signaling	[76]
Novel Proteins	PTF1ɑ (Pancreas Specific Transcription Factor, 1ɑ)	Specifically expressed in the brain and pancreas, Important role in early specification of all exocrine progenitor	[47, 48, 57, 77]
	MIST-1 (BHA15) (Muscle, Intestine and Stomach expression-1)	Expressed in acinar cells in the pancreas, Known to affect the differentiation and morphology of exocrine cells especially acinar cells	[49. 50]
	ASCL3 (Achaete-Scute Complex homolog 3)	Regulate cell fate specification and differentiation, Contribute to the maintenance of mature salivary glands, Localized in the ductal cells of salivary gland	[51.^52^]

Group6: differentially expressed salivary gland transcription factors detected by iTRAQ and 2-DE, Novel Proteins: newly induced salivary gland transcription factors in differentiated MSCs.

## Discussion

A variety of approaches have been applied to protect SG from radiation therapy-induced damage or from inflammatory changes caused by autoimmune SjS. These approaches include intensity-modulated radiation therapy (IMRT) to minimize the amount of radiation to normal surrounding tissue during radiotherapy [26], pharmacological interventions such as sialagogues or radical scavengers to minimize radiation-induced damage, or use of immunosuppresants [27]. However, these approaches have limitations in restoring or replacing previously damaged tissues. Biological tools such as gene transfer [28] or cell-based methodologies [29–31] appear to be more promising with respect to functional restoration. Rapid progress in the field of regenerative medicine has given new hope to treat and even cure diverse types of disease and/or disorders [32–41].

Resident stem cells or MSCs are undifferentiated cells found in adults throughout life [42] and possess the ability to sustain population of cells in organ and maintain tissue remodeling/repair [43]. Optimization of adult stem cell procedures for regenerative medicine is requisite to overcome some of the challenges for developing stem cell-based therapies. However, to date, the molecular mechanisms underlying stem cell differentiation, especially for MSC differentiation into SECs, has not been adequately explored. In our previous study, we confirmed expression of SEC marker proteins, such as AMY, M3R, AQP5, and CK19 in co-cultured mouse BM-MSCs [9]. Therefore, to understand and establish a molecular blueprint of MSC differentiation for the first time, we utilized our *in vitro* co-culture system where BM-MSCs were incubated with isolated pSGCs from mice for high-throughput quantitative proteome analyses.

Reproducibility of our co-culture results in Fig 1 in our current study reaffirms that soluble factors derived from pSGCs can stimulate BM-MSC-conversion into SECs via paracrine stimulation *in vitro*. Of note, a cell number ratio of 1:6 (mMSCs to pSGCs) was critical to produce the most consistent results in both studies with co-culture. Maria *et al*. used a co-culture cell ratio of 1:3 (hMSCs to hSGCs) to favor human MSC differentiation [44]. Another study has shown that different cell number ratios between articular chondrocytes and MSCs affect chondrogenic differentiation of MSCs and alter cartilage matrix formation in cartilage tissue engineering [45, 46]. Although fine differences of co-culture with different ratios of cells among the laboratories have not been compared or established, it is clear that a feeder cell to stem cell ratio is critical in a co-culture system to ensure consistent results and reproducibility of MSC transdifferentiation *in vitro*.

Quantitative proteomics have proven to be a useful technique for investigation of molecular mechanisms in stem cell studies. iTRAQ is currently the most widely used method for high-throughput protein quantitation, allowing simultaneous quantitation of multiple biological samples. Therefore, we profiled the prospective proteome of differentiated MSCs by iTRAQ accompanied by the utilization of bioinformatics tools. In our co-culture system, roughly four times more number of proteins was upregulated than downregulated in differentiating MSCs (Fig 2A). These upregulated proteins were shown to be related to certain functions, such as apoptosis, cellular, and developmental processes while downregulated proteins were more related to the processes of metabolism or biological adhesion (Fig 3).

In addition, we identified that newly induced or suppressed proteins detected only in differentiated MSCs during co-culture. We hypothesize those novel proteins may play an essential role in signaling cascade that is critical in lineage-specific differentiation. Our data analyses indicate that these novel proteins are also involved in various functions especially in developmental processes (Fig 4B). More specifically, 9 out of 27 proteins (17%), such as ADSV, DC1L1, MIST-1 (BHA15), CK19 (K1C19), PTF1α, COIA1, ADHX, ASCL3 and STAT3, were categorized into development-associated proteins (Fig 4B). Among them, 5 proteins (STAT3, ASCL3, MIST-1 and PTF1α) are known to act as TFs. As summarized in Table 2, Ptf1α is a well-known TF in pancreatic cell differentiation and involved in maturation of exocrine progenitor cells [47, 48]. Mist-1 (Bha15) acts as a regulator of differentiation and morphogenesis of exocrine cells [49, 50] while Ascl3 detected in SG ductal cell is known to play an important role in SG maturation [51, 52]. As indicated in Fig 4A, we also identified several SEC marker proteins in the group of newly induced proteins in co-culture, such as AQP5, M3R, AMY, and CK19, which confirmed our previous immunocytochemistry findings [9].

Surprisingly, publications reporting the expression of Ptf1α in the mouse salivary glands were completely absent until our recent findings. As we detected Ptf1α by iTRAQ in differentiating MSCs, we hypothesized that Ptf1α may play a role in developing SGs as well. The detection of Ptf1α and Mist-1(Bha15) expression in the SG of young C56BL/6 mice in our study supported our hypothesis. iTRAQ data were further verified in independent batches of co-cultured mMSCs by western blotting (Fig 5B). As the name of the molecule, pancreas transcription factor 1 α, specified, Ptf1α has been extensively studied in the field of pancreas regeneration. Therefore, presence of Ptf1α in the co-cultured MSCs and mouse SG may imply that Ptf1α has a previously unrecognized role in SG stem cell differentiation and/or glandular development, which warrants further exploration. Furthermore, we detected two different protein bands of Mist-1 in mouse SG and in the differentiated MSCs (Fig 5). We presume that processing of different isoforms or post-translational modifications of Mist-1 may occur during SG development or MSC differentiation, which requires further investigation.

In the field of SG regeneration, understanding how synergistic interactions among novel and differentially expressed proteins can affect stem cell competency for lineage determination is vital for controlling stem cell fate. Our current data set a foundation for the rationale that identifying and expressing key TFs in stem cells may direct the generation of functional SECs. Therefore, we selected six TFs of interest for future investigation, namely Tcf3, Hmg20B, Ankrd56, Ptf1α, Ascl3(Sgn-1), and Mist-1 (Bha15) (red circles in Fig 6 and proteins listed in Table 2) as critical TFs, based on both 2-DE and iTRAQ analyses (Fig 2B). The putative roles of these six proteins known in literature or in database are summarized in Table 2.

In brief, Tcf3, which is known to be important in exiting the self-renewal stage to initiate cell type-specific differentiation, directly regulates Mist-1 [53, 54]. It also regulates other molecules involved in mammalian cell development, such as Myf1, Myod1, Myf6, Bmp2, Bmp4, Jun, and Atf2 (Fig 6). Interestingly, Myod1 protein involved in developmental process can directly modulate expression and function of Hdac1 protein Hmg20b. Ptf1α, which was studied exclusively in the pancreas, is known to be critical in pancreatic exocrine lineage determination as mentioned in Table 2. In addition, Ptf1α is known to promote three Notch molecules (Notch1, 2 and 3) in organ differentiation [55–57]. Furthermore, Ankrd56 regulates cell fate through notch signaling as a cellular regulator. Ascl3 was identified to regulate cell fate specification and differentiation with bmp family and protein expression was detected in the duct cells of salivary glands [51, 52, 58]. Our current analyses provide complex, yet important insight into the underlying molecular footprint of MSC-to-SEP conversion. Further investigations on functional roles of these TFs in MSC differentiation will warrant rapid progress in this field of SG regeneration.

## Conclusion

Cell fate of stem cells is believed to be dependent on both internal and external signals [59, 60]. The external signals are provided from the surrounding microenvironment via either the direct cell-cell interaction or soluble bioactive factors released in SGs during repair and/or regeneration processes [61–63]. Radiation to the glands or inflammatory changes in the SjS SG creates an undesirable microenvironment [64–68] or absence of vital inducing signals, consequently hindering proper MSC conversion as a result [69]. Our study aims to overcome the challenge of inadequate environmental cues in the damaged SG by identifying key internal TFs and ultimately reprogramming MSCs prior to transplantation.

We quantified the proteome in co-cultured BM-MSCs by iTRAQ-2D-LC-MS/MS and identified differentially expressed/newly induced or suppressed TFs that may be critical in MSC conversion into SEC. For the first time, we generated a TF regulatory network that may be active during the differentiation process of MSCs based on database search and extensive literature search. Identification of intrinsic ques for MSC differentiation will allow MSC conversion to be less dependent on external signals that are most likely deficient in the severely damaged SGs. Our current approach involving viral vectors that express 6TFs of interest will address whether these key TFs are indeed pivotal in triggering and driving MSCs into functional SECs. It is our hope that discovery of internal signals (TFs or STFs) for MSC conversion is instrumental for future research on SG regeneration.

## Supporting information

S1 File Quantitative analysis of differentially expressed proteins in co-cultured MSCs. Fig A. Isolated protein lysates from three biological replicates (set #1, set #2, and set #3) were subject to 8-plex iTRAQ-LC-MS/MS for protein identification and quantification of co-cultured MSCs. Overall, 1,844 distinct proteins were identified and quantified as differentially expressed proteins by 95% cut-off value from the three sets. Moreover, 1297 proteins in parenthesis indicate at least three peptides matched against the Uniprot database. Fig B. 1844 identified proteins were tested using different expression cut-off values (±20%, 30% or 40% in relative intensity). Of 1844, 280 proteins (15.19%) proteins were finally selected by using a 30% cut-off value with p < 0.05. Dots highlighted in red indicate upregulated proteins that passed the 30% cut-off value with p < 0.05 and green dots indicate down-regulated proteins.
(TIF)

S2 File Tables of differentially expressed proteins. Table A. List of the 280 differentially expressed proteins. Table B. List of proteins in Fig 4A categorized into six groups based on their expression patterns during co-culture.
(DOCX)

## Abbreviations

AMY1: Alpha-amylase
AQP5: Aquaporin-5
mBM-MSC: Mouse bone marrow-derived mesenchymal stem cell
CK19: Cytokeratin-19
IMRT: Intensity-modulated radiation therapy
IR: Irradiation
iTRAQ: Isobaric tag for relative and absolute quantitation
LC-MS/MS: Liquid chromatography-tandem mass spectrometry
LG: Lacrimal gland
M3R: Muscarinic type 3 receptor
SG: Salivary gland
pSGC: Primary salivary gland cell
SEC: Salivary gland epithelial cell
SEP: Salivary epithelial precursors
SjS: Sjögren’s syndrome
STF: Salivary transcription factor
TFRF: Transcription regulatory factor
TCF3: Transcription factor E2 alpha
HMG20b: High mobility group protein 20B
ANKRD56: Ankyrin repeat domain-containing protein 56
PTF1α: Pancreas specific transcription factor 1 alpha
MIST-1: Muscle, intestine and stomach expression-1 (alternative name; BHA15)
ASCL3: Achaete-scute complex homolog 3, (alternative name; SGN-1)
