# Supplementary figures and images for: Uncovering stem cell differentiation factors for salivary gland regeneration by quantitative analysis of differential proteomes
Source: PLoS One. 2017 Feb 3;12(2):e0169677. doi: 10.1371/journal.pone.0169677 (PMC5291466; doi:10.1371/journal.pone.0169677)

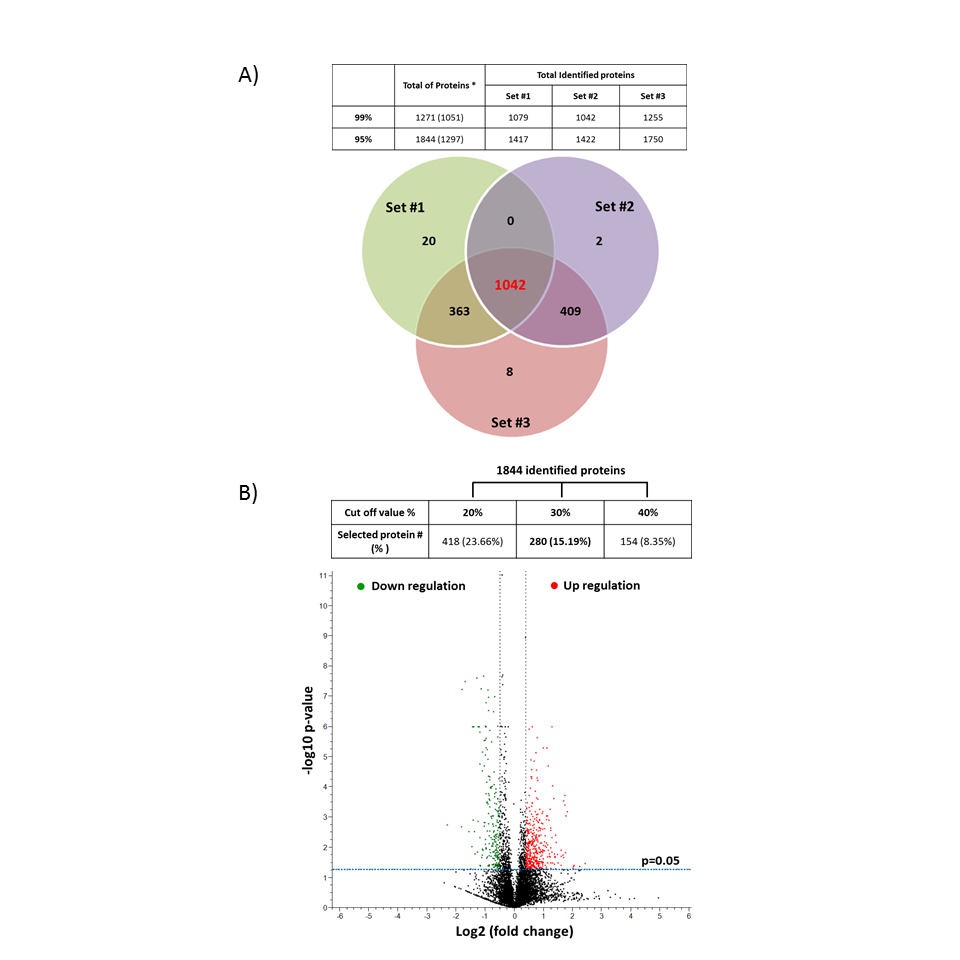

Supplement: S1 File — Fig A. Isolated protein lysates from three biological replicates (set #1, set #2, and set #3) were subject to 8-plex iTRAQ-LC-MS/MS for protein identification and quantification of co-cultured MSCs. Overall, 1,844 distinct proteins were identified and quantified as differentially expressed proteins by 95% cut-off value from the three sets. Moreover, 1297 proteins in parenthesis indicate at least three peptides matched against the Uniprot database. Fig B. 1844 identified proteins were tested using different expression cut-off values (±20%, 30% or 40% in relative intensity). Of 1844, 280 proteins (15.19%) proteins were finally selected by using a 30% cut-off value with p < 0.05. Dots highlighted in red indicate upregulated proteins that passed the 30% cut-off value with p < 0.05 and green dots indicate down-regulated proteins. (TIF) [file pone.0169677.s001.tif]
