# Supplementary material for: Uncovering stem cell differentiation factors for salivary gland regeneration by quantitative analysis of differential proteomes
Source: PLoS One. 2017 Feb 3;12(2):e0169677. doi: 10.1371/journal.pone.0169677 (PMC5291466; doi:10.1371/journal.pone.0169677)
Supplement: S2 File — Table A. List of the 280 differentially expressed proteins. Table B. List of proteins in Fig 4A categorized into six groups based on their expression patterns during co-culture. (DOCX) [file pone.0169677.s002.docx]

Table A. List of the 280 differentially expressed proteins

| Protein  ID | Accession  Number | Annotation | Average Fold Change | | | |  | P-valule | | | |
| --- | --- | --- | --- | --- | --- | --- | --- | --- | --- | --- | --- |
|  |  |  | 1day | 3day | 5day | 7day |  | 1day | 3day | 5day | 7day |
| CP-1 | tr\|Q8C153\|Q8C153 | Putative uncharacterized protein | 0.9137 | 0.9412 | 1.3187 | 1.3477 |  | 0.07449 | 0.11567 | 0.00002 | 0.00111 |
| CP-2 | sp\|Q9Z104\|HMGP20B | SWI/SNF-related matrix-associated actin-dependent regulator of chromatin subfamily E member 1-HMG20B | 1.3039 | 1.6348 | 1.6137 | 1.6463 |  | 0.06022 | 0.09721 | 0.00001 | 0.00144 |
| CP-3 | tr\|Q8BMA8\|Q8BMA8 | Putative uncharacterized protein | 0.9064 | 0.9304 | 1.3513 | 1.3858 |  | 0.06392 | 0.09308 | 0.00001 | 0.00086 |
| CP-4 | sp\|P56480\|ATPB | ATP synthase subunit beta, mitochondrial | 1.3191 | 1.3777 | 1.6368 | 1.5821 |  | 0.00002 | 0.00006 | 0.00000 | 0.00000 |
| CP-5 | sp\|P05064\|ALDOA | Fructose-bisphosphate aldolase A | 1.0865 | 1.1443 | 1.2899 | 1.3015 |  | 0.00512 | 0.00232 | 0.00000 | 0.00000 |
| CP-6 | sp\|Q03265\|ATPA | ATP synthase subunit alpha, mitochondrial | 1.0834 | 1.0864 | 1.4579 | 1.3582 |  | 0.17555 | 0.18449 | 0.00000 | 0.00007 |
| CP-7 | sp\|P10107\|ANXA1 | Annexin A1 | 1.4173 | 1.5047 | 2.0506 | 1.9827 |  | 0.00005 | 0.00001 | 0.00000 | 0.00000 |
| CP-8 | sp\|P15806\|TCF3 | Transcription factor E2-alpha | 1.3321 | 1.5285 | 1.5586 | 1.5200 |  | 0.00001 | 0.00001 | 0.00000 | 0.00000 |
| CP-9 | sp\|P09103\|PDIA1 | Protein disulfide-isomerase | 1.4898 | 1.4982 | 2.1266 | 2.0236 |  | 0.00000 | 0.00003 | 0.00000 | 0.00000 |
| CP-10 | sp\|P06151\|LDHA | L-lactate dehydrogenase A chain | 1.2559 | 1.3686 | 1.2757 | 1.2755 |  | 0.00250 | 0.00022 | 0.00124 | 0.00151 |
| CP-11 | sp\|Q68FD5\|CLH1 | Clathrin heavy chain 1 | 1.2194 | 1.3188 | 1.2834 | 1.2449 |  | 0.00172 | 0.00000 | 0.00001 | 0.00122 |
| CP-12 | sp\|P08249\|MDHM | Malate dehydrogenase, mitochondrial | 1.3499 | 1.3113 | 1.4146 | 1.3389 |  | 0.00040 | 0.00108 | 0.00003 | 0.00078 |
| CP-13 | sp\|Q922F4\|TBB6 | Tubulin beta-6 chain | 1.6340 | 1.5850 | 1.0821 | 1.5248 |  | 0.09179 | 0.01060 | 0.00403 | 0.00020 |
| CP-14 | sp\|P17751\|TPIS | Triosephosphate isomerase | 1.1000 | 1.1534 | 1.4409 | 1.4343 |  | 0.02935 | 0.02005 | 0.00000 | 0.00001 |
| CP-15 | sp\|Q99KI0\|ACON | Aconitate hydratase, mitochondrial | 1.2480 | 1.2210 | 1.6707 | 1.5674 |  | 0.00016 | 0.00212 | 0.00000 | 0.00004 |
| CP-16 | spP48036\|ANXA5 | Annexin A5 | 1.2960 | 1.2978 | 1.5204 | 1.5727 |  | 0.00515 | 0.00204 | 0.00001 | 0.00001 |
| CP-17 | tr\|Q3UGY5\|Q3UGY5 | Putative uncharacterized protein | 0.4029 | 0.4246 | 0.4853 | 0.5317 |  | 0.00000 | 0.00000 | 0.00000 | 0.00000 |
| CP-18 | tr\|Q3UH17\|Q3UH17 | Putative uncharacterized protein | 0.4233 | 0.4347 | 0.4870 | 0.5202 |  | 0.00000 | 0.00000 | 0.00000 | 0.00000 |
| CP-19 | sp\|P97429\|ANXA4 | Annexin A4 | 2.3848 | 2.7696 | 2.4961 | 2.4570 |  | 0.00000 | 0.00000 | 0.00000 | 0.00000 |
| CP-20 | tr\|A8DUK4\|A8DUK4 | Beta-globin | 3.6177 | 3.0447 | 2.8251 | 2.9435 |  | 0.00000 | 0.00000 | 0.00056 | 0.00016 |
| CP-21 | sp\|Q8VHX6-2\|FLNC | Isoform 2 of Filamin-C | 0.7848 | 0.8059 | 0.7123 | 0.6699 |  | 0.00002 | 0.00005 | 0.00000 | 0.00000 |
| CP-22 | sp\|P14211\|CALR | Calreticulin | 1.3137 | 1.3474 | 1.4447 | 1.3923 |  | 0.00012 | 0.00080 | 0.00000 | 0.00000 |
| CP-23 | sp\|Q60605-2\|MYL6 | Isoform Smooth muscle of Myosin light polypeptide 6 | 0.7320 | 0.6627 | 1.5203 | 1.4813 |  | 0.00001 | 0.00000 | 0.00943 | 0.00060 |
| CP-24 | sp\|P58771-2\|TPM1 | Isoform 2 of Tropomyosin alpha-1 chain | 0.8410 | 0.8405 | 0.5442 | 0.5394 |  | 0.00001 | 0.00000 | 0.00000 | 0.00000 |
| CP-25 | sp\|P05202\|AATM | Aspartate aminotransferase, mitochondrial | 1.3815 | 1.4145 | 1.5505 | 1.5191 |  | 0.00000 | 0.00002 | 0.00000 | 0.00000 |
| CP-26 | tr\|Q61344\|Q61344 | Beta-tropomyosin | 0.5501 | 0.5973 | 0.6024 | 0.5854 |  | 0.00000 | 0.00000 | 0.00000 | 0.00000 |
| CP-27 | sp\|Q9D8E6\|RL4 | 60S ribosomal protein L4 | 1.7110 | 1.6083 | 1.3804 | 1.4536 |  | 0.00000 | 0.00002 | 0.00056 | 0.00043 |
| CP-28 | sp\|Q61699-2\|HS105 | Isoform HSP105-beta of Heat shock protein 105 kDa | 0.6299 | 0.6214 | 0.5712 | 0.5472 |  | 0.00000 | 0.00000 | 0.00001 | 0.00000 |
| CP-29 | tr\|E9Q450\|E9Q450 | Tropomyosin alpha-1 chain | 0.5160 | 0.5170 | 0.5666 | 0.5631 |  | 0.00000 | 0.00000 | 0.00000 | 0.00000 |
| CP-30 | sp\|Q60605\|MYL6 | Myosin light polypeptide 6 | 0.7618 | 0.7690 | 1.5345 | 1.4907 |  | 0.00000 | 0.00000 | 0.00484 | 0.00040 |
| CP-31 | tr\|G5E8R1\|G5E8R1 | Tropomyosin 1, alpha, isoform CRA_j | 0.9609 | 0.9525 | 0.5542 | 0.5509 |  | 0.00022 | 0.00000 | 0.00000 | 0.00000 |
| CP-32 | sp\|P54071\|IDHP | Isocitrate dehydrogenase [NADP], mitochondrial | 1.1514 | 1.1378 | 1.3565 | 1.3345 |  | 0.02140 | 0.06234 | 0.00007 | 0.00002 |
| CP-33 | sp\|P99029-2\|PRDX5 | Isoform Cytoplasmic+peroxisomal of Peroxiredoxin-5, mitochondrial | 1.5962 | 1.4926 | 1.9418 | 1.8519 |  | 0.03051 | 0.03264 | 0.00002 | 0.00021 |
| CP-34 | sp\|P58774-2\|TPM2 | Isoform 2 of Tropomyosin beta chain | 0.5656 | 0.5983 | 0.5865 | 0.5795 |  | 0.00000 | 0.00000 | 0.00000 | 0.00000 |
| CP-35 | sp\|P18760\|COF1 | Cofilin-1 | 0.7055 | 0.6695 | 0.9771 | 0.9021 |  | 0.00052 | 0.00016 | 0.00479 | 0.03131 |
| CP-36 | sp\|P26443\|DHE3 | Glutamate dehydrogenase 1, mitochondrial | 1.3989 | 1.4211 | 1.5411 | 1.5816 |  | 0.00475 | 0.00875 | 0.00128 | 0.00185 |
| CP-37 | sp\|Q3UPL0\|SC31A | Protein transport protein Sec31A | 1.2722 | 1.3464 | 1.2951 | 1.2439 |  | 0.00038 | 0.00030 | 0.00080 | 0.00001 |
| CP-38 | tr\|G5E8R2\|G5E8R2 | Tropomyosin 1, alpha, isoform CRA_k | 0.5385 | 0.5323 | 0.5820 | 0.5804 |  | 0.00000 | 0.00000 | 0.00000 | 0.00000 |
| CP-39 | sp\|P62806\|H4 | Histone H4 | 1.5887 | 1.6570 | 1.3000 | 1.5123 |  | 0.00081 | 0.00030 | 0.00237 | 0.00014 |
| CP-40 | sp\|P70333\|HNRH2 | Heterogeneous nuclear ribonucleoprotein H2 | 1.1403 | 1.2795 | 1.3711 | 1.3354 |  | 0.12893 | 0.06083 | 0.00549 | 0.00611 |
| CP-41 | sp\|P10605\|CATB | Cathepsin B | 1.3919 | 1.5922 | 1.9867 | 2.1151 |  | 0.01435 | 0.02408 | 0.00047 | 0.00105 |
| CP-42 | sp\|Q91VI7\|RINI | Ribonuclease inhibitor | 1.4556 | 1.4694 | 1.9839 | 1.8487 |  | 0.01691 | 0.01833 | 0.00013 | 0.00003 |
| CP-43 | sp\|P47738\|ALDH2 | Aldehyde dehydrogenase, mitochondrial | 1.0957 | 1.2272 | 1.4898 | 1.4120 |  | 0.18341 | 0.03901 | 0.01554 | 0.00401 |
| CP-44 | sp\|Q61553\|FSCN1 | Fascin | 0.7708 | 0.7288 | 0.4694 | 0.5933 |  | 0.01695 | 0.01110 | 0.00336 | 0.00992 |
| CP-45 | sp\|P13020-2\|GELS | Isoform 2 of Gelsolin | 1.3419 | 1.4316 | 1.4519 | 1.3742 |  | 0.00004 | 0.00005 | 0.00001 | 0.00046 |
| CP-46 | sp\|Q7TMK9-2\|HNRPQ | Isoform 2 of Heterogeneous nuclear ribonucleoprotein Q | 0.5692 | 0.6002 | 0.8336 | 0.7629 |  | 0.00026 | 0.00050 | 0.01414 | 0.00367 |
| CP-47 | sp\|Q99LC5\|ETFA | Electron transfer flavoprotein subunit alpha, mitochondrial | 1.1090 | 1.1417 | 1.5650 | 1.5569 |  | 0.00746 | 0.00736 | 0.00089 | 0.00014 |
| CP-48 | sp\|P07724\|ALBU | Serum albumin | 1.4490 | 1.4061 | 0.9372 | 1.0654 |  | 0.00015 | 0.00961 | 0.46352 | 0.42571 |
| CP-49 | tr\|Q6ZWQ9\|Q6ZWQ9 | MCG5400 | 0.5887 | 0.6144 | 0.8492 | 0.6722 |  | 0.00000 | 0.00000 | 0.00174 | 0.00000 |
| CP-50 | sp\|P27659\|RL3 | 60S ribosomal protein L3 | 1.4041 | 1.3254 | 1.1316 | 1.0812 |  | 0.00003 | 0.00015 | 0.00951 | 0.02391 |
| CP-51 | sp\|Q8VDN2\|AT1A1 | Sodium/potassium-transporting ATPase subunit alpha-1 | 0.9797 | 1.0623 | 1.2173 | 1.3369 |  | 0.00072 | 0.00272 | 0.04248 | 0.00025 |
| CP-52 | tr\|Q99N15\|Q99N15 | 17beta-hydroxysteroid dehydrogenase type 10/short chain L-3-hydroxyacyl-CoA dehydrogenase | 1.0856 | 1.2375 | 1.3101 | 1.0443 |  | 0.11000 | 0.02149 | 0.01470 | 0.04979 |
| CP-53 | sp\|Q9CZU6\|CISY | Citrate synthase, mitochondrial | 1.2411 | 1.2348 | 1.4285 | 1.3319 |  | 0.00724 | 0.00997 | 0.00001 | 0.00019 |
| CP-54 | sp\|Q3THE2\|ML12B | Myosin regulatory light chain 12B | 0.6111 | 0.6368 | 0.7895 | 0.6929 |  | 0.00000 | 0.00000 | 0.00000 | 0.00000 |
| CP-55 | sp\|Q99PL5\|RRBP1 | Ribosome-binding protein 1 | 0.9975 | 1.2258 | 1.5958 | 1.7312 |  | 0.00731 | 0.00243 | 0.00004 | 0.00004 |
| CP-56 | tr\|H9BUF4\|H9BUF4_9CRYT | Actin (Fragment) | 0.8192 | 0.7866 | 0.5880 | 0.6314 |  | 0.03482 | 0.02637 | 0.00275 | 0.03240 |
| CP-57 | sp\|O08638-2\|MYH11 | Isoform 2 of Myosin-11 | 0.7889 | 0.7750 | 1.3621 | 1.3365 |  | 0.00006 | 0.00007 | 0.00851 | 0.00472 |
| CP-58 | sp\|P62334\|PRS10 | 26S protease regulatory subunit 10B | 1.1567 | 1.0971 | 1.5444 | 1.2784 |  | 0.22918 | 0.31903 | 0.03977 | 0.15606 |
| CP-59 | sp\|Q8BFR5-2\|EFTU | Isoform 2 of Elongation factor Tu, mitochondrial | 1.2662 | 1.2087 | 1.5504 | 1.3266 |  | 0.00058 | 0.00090 | 0.00006 | 0.00137 |
| CP-60 | sp\|P35700\|PRDX1 | Peroxiredoxin-1 | 1.2877 | 1.2918 | 1.3087 | 1.4094 |  | 0.00230 | 0.00705 | 0.00004 | 0.00023 |
| CP-61 | tr\|Q91XI2\|Q91XI2 | Catalase | 2.2034 | 2.4726 | 2.1272 | 2.5644 |  | 0.19707 | 0.00242 | 0.00064 | 0.00052 |
| CP-62 | sp\|P14152\|MDHC | Malate dehydrogenase, cytoplasmic | 1.5062 | 1.5062 | 1.4971 | 1.4420 |  | 0.08307 | 0.08307 | 0.00234 | 0.00800 |
| CP-63 | sp\|Q9DCW4\|ETFB | Electron transfer flavoprotein subunit beta | 1.1376 | 1.0127 | 1.4205 | 1.3566 |  | 0.04691 | 0.11361 | 0.01082 | 0.00352 |
| CP-64 | sp\|P24270\|CATA | Catalase | 1.0219 | 2.1974 | 2.3274 | 2.5442 |  | 0.12289 | 0.00327 | 0.00069 | 0.00056 |
| CP-65 | tr\|Q3TVZ1\|Q3TVZ1 | Catalase | 1.9160 | 2.1852 | 2.2579 | 2.4494 |  | 0.15458 | 0.00177 | 0.00457 | 0.00576 |
| CP-66 | sp\|Q8BMS1\|ECHA | Trifunctional enzyme subunit alpha, mitochondrial | 1.1493 | 1.2423 | 1.5758 | 1.5204 |  | 0.30905 | 0.20930 | 0.00447 | 0.02288 |
| CP-67 | sp\|Q60710\|SAMH1 | SAM domain and HD domain-containing protein 1 | 2.0053 | 2.0784 | 1.8780 | 1.8852 |  | 0.03168 | 0.01017 | 0.00392 | 0.00825 |
| CP-68 | tr\|Q3UJ44\|Q3UJ44 | Putative uncharacterized protein | 1.1725 | 1.2281 | 1.6247 | 1.4642 |  | 0.09138 | 0.05273 | 0.00476 | 0.00638 |
| CP-69 | tr\|F6Q404\|F6Q404 | Thioredoxin (Fragment) | 1.5866 | 1.5532 | 1.3505 | 1.6380 |  | 0.03974 | 0.07433 | 0.01560 | 0.01482 |
| CP-70 | sp\|P97855\|G3BP1 | Ras GTPase-activating protein-binding protein 1 | 0.7099 | 0.7281 | 0.7074 | 0.6226 |  | 0.01157 | 0.01211 | 0.01960 | 0.00693 |
| CP-71 | sp\|P18242\|CATD | Cathepsin D | 1.4354 | 1.4718 | 2.2389 | 2.2687 |  | 0.00008 | 0.00043 | 0.00002 | 0.00034 |
| CP-72 | sp\|P63028\|TCTP | Translationally-controlled tumor protein | 0.7449 | 0.6515 | 0.6118 | 0.6776 |  | 0.01818 | 0.00439 | 0.00009 | 0.00005 |
| CP-73 | sp\|P46935\|NEDD4 | E3 ubiquitin-protein ligase NEDD4 | 0.7219 | 0.6440 | 0.8054 | 0.7305 |  | 0.01054 | 0.00237 | 0.00509 | 0.00736 |
| CP-74 | sp\|P62827\|RAN | GTP-binding nuclear protein Ran | 0.7190 | 0.6978 | 0.7773 | 0.8019 |  | 0.00664 | 0.00476 | 0.00702 | 0.02232 |
| CP-75 | sp\|P14824\|ANXA6 | Annexin A6 | 0.7457 | 0.7435 | 1.3791 | 1.2622 |  | 0.05517 | 0.05289 | 0.00980 | 0.04160 |
| CP-76 | tr\|Q9CY06\|Q9CY06 | Putative uncharacterized protein | 3.9836 | 2.8340 | 1.7271 | 1.5256 |  | 0.01485 | 0.04210 | 0.18551 | 0.18592 |
| CP-77 | sp\|Q9QZE5\|COPG1 | Coatomer subunit gamma-1 | 1.4641 | 1.5768 | 1.2359 | 1.2351 |  | 0.08611 | 0.03286 | 0.21538 | 0.12039 |
| CP-78 | sp\|Q05920\|PYC | Pyruvate carboxylase, mitochondrial | 1.4430 | 1.4461 | 2.5347 | 2.4287 |  | 0.03311 | 0.03375 | 0.00013 | 0.00025 |
| CP-79 | tr\|Q3U505\|Q3U505 | Putative uncharacterized protein (Fragment) | 1.6177 | 1.5552 | 1.1179 | 1.3125 |  | 0.01789 | 0.02921 | 0.37773 | 0.06804 |
| CP-80 | sp\|Q64674\|SPEE | Spermidine synthase | 1.3797 | 1.3797 | 1.4044 | 1.3558 |  | 0.10365 | 0.10365 | 0.04235 | 0.05197 |
| CP-81 | tr\|E9QA15\|E9QA15 | Protein Cald1 | 0.6071 | 0.6887 | 0.6236 | 0.6105 |  | 0.00000 | 0.00000 | 0.00000 | 0.00000 |
| CP-82 | tr\|E9QA16\|E9QA16 | Protein Cald1 | 0.5140 | 0.6112 | 0.5624 | 0.5612 |  | 0.00000 | 0.00000 | 0.00000 | 0.00000 |
| CP-83 | sp\|Q8R0W0\|EPIPL | Epiplakin | 1.1820 | 1.1432 | 1.4750 | 1.4093 |  | 0.01550 | 0.14986 | 0.02692 | 0.01037 |
| CP-84 | sp\|Q8R1F1\|NIBL1 | Niban-like protein 1 | 1.2743 | 1.3351 | 1.3546 | 1.2520 |  | 0.00959 | 0.00502 | 0.08198 | 0.06665 |
| CP-85 | sp\|Q99JY0\|ECHB | Trifunctional enzyme subunit beta, mitochondrial | 1.2986 | 1.4124 | 1.7620 | 1.5143 |  | 0.02042 | 0.02177 | 0.00002 | 0.00023 |
| CP-86 | sp\|Q9DCD0\|6PGD | 6-phosphogluconate dehydrogenase, decarboxylating | 1.1847 | 1.2427 | 1.2855 | 1.3457 |  | 0.02042 | 0.00746 | 0.00874 | 0.00364 |
| CP-87 | sp\|P24369\|PPIB | Peptidyl-prolyl cis-trans isomerase B | 1.3471 | 1.4048 | 1.5498 | 1.4166 |  | 0.00009 | 0.00000 | 0.00000 | 0.00001 |
| CP-88 | sp\|P15626\|GSTM2 | Glutathione S-transferase Mu 2 | 1.7736 | 1.7736 | 1.7533 | 1.7284 |  | 0.00215 | 0.00215 | 0.00000 | 0.00001 |
| CP-89 | sp\|P34884\|MIF | Macrophage migration inhibitory factor | 1.0806 | 1.0554 | 1.4100 | 1.2162 |  | 0.24215 | 0.37615 | 0.02530 | 0.04274 |
| CP-90 | tr\|Q3UZH2\|Q3UZH2 | Putative uncharacterized protein | 0.8791 | 1.0996 | 0.8130 | 0.6987 |  | 0.10504 | 0.14053 | 0.00119 | 0.00034 |
| CP-91 | sp\|Q93092\|TALDO | Transaldolase | 1.1124 | 1.1124 | 1.3282 | 1.2761 |  | 0.35510 | 0.35510 | 0.00329 | 0.01660 |
| CP-92 | sp\|P47911\|RL6 | 60S ribosomal protein L6 | 2.1040 | 1.8907 | 1.5771 | 1.6757 |  | 0.00173 | 0.00453 | 0.01313 | 0.06278 |
| CP-93 | tr\|Q9Z1R9\|Q9Z1R9 | MCG124046 | 1.4395 | 2.6827 | 1.4628 | 1.6971 |  | 0.04883 | 0.05962 | 0.04454 | 0.10137 |
| CP-94 | sp\|O08749\|DLDH | Dihydrolipoyl dehydrogenase, mitochondrial | 1.2615 | 1.3259 | 1.5057 | 1.4403 |  | 0.01278 | 0.00415 | 0.01432 | 0.01360 |
| CP-95 | sp\|Q01149\|CO1A2 | Collagen alpha-2(I) chain | 0.5140 | 0.5140 | 0.6691 | 0.6540 |  | 0.02192 | 0.02192 | 0.00522 | 0.00304 |
| CP-96 | sp\|Q9D1Q6\|ERP44 | Endoplasmic reticulum resident protein 44 | 1.1668 | 1.4443 | 1.5233 | 1.5470 |  | 0.01041 | 0.00268 | 0.00753 | 0.00695 |
| CP-97 | sp\|P28656\|NP1L1 | Nucleosome assembly protein 1-like 1 | 0.5822 | 0.8026 | 0.5380 | 0.5436 |  | 0.04946 | 0.03307 | 0.13581 | 0.04107 |
| CP-98 | sp\|Q8BWT1\|THIM | 3-ketoacyl-CoA thiolase, mitochondrial | 1.5806 | 1.7148 | 1.7432 | 1.9473 |  | 0.00529 | 0.00222 | 0.00960 | 0.00366 |
| CP-99 | sp\|P52293\|IMA2 | Integrin beta-1 | 0.8429 | 0.7455 | 0.7755 | 0.6954 |  | 0.01143 | 0.00204 | 0.04430 | 0.00158 |
| CP-100 | sp\|P19157\|GSTP1 | Glutathione S-transferase P 1 | 1.1380 | 1.2711 | 1.6192 | 1.3401 |  | 0.08568 | 0.01161 | 0.04717 | 0.16032 |
| CP-101 | sp\|P50543\|S10AB | Protein S100-A11 | 1.2051 | 1.3526 | 1.4654 | 1.4885 |  | 0.00546 | 0.00050 | 0.00006 | 0.00008 |
| CP-102 | sp\|Q08093\|CNN2 | Calponin-2 | 0.6741 | 0.6536 | 0.7522 | 0.6542 |  | 0.00020 | 0.00011 | 0.00194 | 0.00063 |
| CP-103 | sp\|P09055\|ITB1 | Importin subunit alpha-2 | 1.4969 | 1.4667 | 1.5033 | 1.3199 |  | 0.16870 | 0.29224 | 0.00116 | 0.00103 |
| CP-104 | sp\|Q9R1J0\|NSDHL | Sterol-4-alpha-carboxylate 3-dehydrogenase, decarboxylating | 1.9260 | 1.9260 | 1.4881 | 1.7239 |  | 0.01733 | 0.01733 | 0.19848 | 0.01478 |
| CP-105 | sp\|P16110\|LEG3 | Galectin-3 | 0.9247 | 1.0989 | 1.3831 | 1.4346 |  | 0.15912 | 0.02664 | 0.00315 | 0.00098 |
| CP-106 | sp\|Q9DB77\|QCR2 | Cytochrome b-c1 complex subunit 2, mitochondrial | 1.5830 | 1.6833 | 1.4974 | 1.5200 |  | 0.00426 | 0.02598 | 0.00245 | 0.05894 |
| CP-107 | sp\|Q4KML4\|ABRAL | Costars family protein ABRACL | 0.5017 | 0.8313 | 0.8180 | 0.9817 |  | 0.00487 | 0.01432 | 0.70373 | 0.91841 |
| CP-108 | sp\|Q9CZ13\|QCR1 | Cytochrome b-c1 complex subunit 1, mitochondrial | 1.5481 | 1.5481 | 1.6779 | 1.4991 |  | 0.03911 | 0.03911 | 0.04221 | 0.01507 |
| CP-109 | sp\|Q6ZWN5\|RS9 | 40S ribosomal protein S9 | 1.4076 | 1.3115 | 1.1007 | 1.0905 |  | 0.00086 | 0.00622 | 0.03310 | 0.02209 |
| CP-110 | sp\|Q07797\|LG3BP | Galectin-3-binding protein | 1.5083 | 1.9875 | 4.0478 | 4.6866 |  | 0.11391 | 0.01644 | 0.04283 | 0.04205 |
| CP-111 | tr\|Q05B38\|Q05B38 | Krt6b protein (Fragment) | 1.3084 | 1.3332 | 3.0879 | 2.4301 |  | 0.02169 | 0.02110 | 0.00932 | 0.00011 |
| CP-112 | sp\|Q64433\|CH10 | 10 kDa heat shock protein, mitochondrial | 0.6476 | 0.7097 | 0.7200 | 0.6776 |  | 0.00018 | 0.00024 | 0.01234 | 0.04608 |
| CP-113 | sp\|P81117\|NUCB2 | Nucleobindin-2 | 1.0990 | 1.2990 | 1.5104 | 1.3155 |  | 0.00706 | 0.00706 | 0.00428 | 0.00292 |
| CP-114 | sp\|Q9D0K2\|SCOT1 | Succinyl-CoA:3-ketoacid coenzyme A transferase 1, mitochondrial | 0.9262 | 0.9165 | 1.2707 | 1.3009 |  | 0.28767 | 0.28510 | 0.00457 | 0.01511 |
| CP-115 | sp\|O08709\|PRDX6 | Peroxiredoxin-6 | 0.9029 | 1.3529 | 1.6968 | 1.2502 |  | 0.06656 | 0.06855 | 0.04426 | 0.00795 |
| CP-116 | sp\|F6ZDS4\|TPR | Nucleoprotein TPR | 1.3409 | 1.3043 | 1.3378 | 1.2508 |  | 0.01071 | 0.00586 | 0.02104 | 0.05556 |
| CP-117 | sp\|P14148\|RL7 | 60S ribosomal protein L7 | 2.3049 | 2.1432 | 1.7090 | 1.9296 |  | 0.00224 | 0.00107 | 0.00012 | 0.00056 |
| CP-118 | sp\|P17047-2\|LAMP2 | Isoform LAMP-2B of Lysosome-associated membrane glycoprotein 2 | 1.4886 | 1.6131 | 1.4462 | 1.7970 |  | 0.00595 | 0.00368 | 0.13498 | 0.00621 |
| CP-119 | sp\|Q8K1J6\|TRNT1 | CCA tRNA nucleotidyltransferase 1, mitochondrial | 1.3665 | 1.3104 | 1.1409 | 1.1566 |  | 0.01452 | 0.02493 | 0.07546 | 0.10160 |
| CP-120 | sp\|Q9D6R2\|IDH3A | Isocitrate dehydrogenase [NAD] subunit alpha, mitochondrial | 1.3584 | 1.3560 | 1.3210 | 1.3822 |  | 0.01214 | 0.01199 | 0.04424 | 0.17852 |
| CP-121 | sp\|P62242\|RS8 | 40S ribosomal protein S8 | 1.3800 | 1.2566 | 1.1359 | 1.1154 |  | 0.00595 | 0.09718 | 0.13088 | 0.08567 |
| CP-122 | tr\|Q3U8W9\|Q3U8W9 | Putative uncharacterized protein | 0.6820 | 0.7251 | 0.6927 | 0.7785 |  | 0.01873 | 0.03600 | 0.23418 | 0.12323 |
| CP-123 | tr\|Q3UKH3\|Q3UKH3 | Putative uncharacterized protein | 1.5964 | 1.5964 | 1.6427 | 1.5955 |  | 0.00436 | 0.00436 | 0.00453 | 0.00618 |
| CP-124 | sp\|Q6PIE5\|AT1A2 | Sodium/potassium-transporting ATPase subunit alpha-2 | 0.9974 | 1.0434 | 1.1470 | 1.4647 |  | 0.00513 | 0.00751 | 0.11151 | 0.00238 |
| CP-125 | sp\|Q61033\|LAP2A | Lamina-associated polypeptide 2, isoforms alpha/zeta | 0.5697 | 0.5652 | 0.7036 | 0.5560 |  | 0.00589 | 0.00587 | 0.01645 | 0.00028 |
| CP-126 | sp\|Q9EPL8\|IPO7 | Importin-7 | 0.8726 | 0.8374 | 0.6782 | 0.6713 |  | 0.27319 | 0.06751 | 0.02087 | 0.01332 |
| CP-127 | tr\|F6UT49\|F6UT49 | Glyceraldehyde-3-phosphate dehydrogenase | 1.1953 | 1.3209 | 1.2273 | 1.3568 |  | 0.14514 | 0.09031 | 0.11203 | 0.04235 |
| CP-128 | tr\|Q8C622\|Q8C622 | Putative uncharacterized protein | 1.6123 | 1.5321 | 0.9664 | 1.4995 |  | 0.02220 | 0.03173 | 0.08625 | 0.02691 |
| CP-129 | sp\|Q8K2B3\|DHSA | Succinate dehydrogenase [ubiquinone] flavoprotein subunit, mitochondrial | 1.3545 | 1.3738 | 1.7247 | 1.5173 |  | 0.03568 | 0.05166 | 0.00204 | 0.00310 |
| CP-130 | sp\|P12787\|COX5A | Cytochrome c oxidase subunit 5A, mitochondrial | 1.1314 | 1.1314 | 1.6027 | 1.4019 |  | 0.31982 | 0.31982 | 0.00378 | 0.03871 |
| CP-131 | sp\|P19253\|RL13A | 60S ribosomal protein L13a | 1.6086 | 1.5000 | 1.3934 | 1.3586 |  | 0.00304 | 0.00540 | 0.00002 | 0.00487 |
| CP-132 | sp\|Q9D051\|ODPB | Pyruvate dehydrogenase E1 component subunit beta, mitochondrial | 1.2438 | 1.2438 | 1.6806 | 1.4890 |  | 0.10467 | 0.10467 | 0.00005 | 0.00040 |
| CP-133 | sp\|Q9R0E2\|PLOD1 | Procollagen-lysine,2-oxoglutarate 5-dioxygenase 1 | 1.7053 | 1.9191 | 1.7206 | 1.6988 |  | 0.00216 | 0.00120 | 0.00605 | 0.00042 |
| CP-134 | sp\|P56391\|CX6B1 | Cytochrome c oxidase subunit 6B1 | 1.6102 | 1.6102 | 1.7184 | 1.4438 |  | 0.05828 | 0.05828 | 0.08645 | 0.03509 |
| CP-135 | sp\|O54724\|PTRF | Polymerase I and transcript release factor | 0.4660 | 0.5971 | 0.8913 | 0.7937 |  | 0.00334 | 0.01019 | 0.14472 | 0.05998 |
| CP-136 | sp\|P19783\|COX41 | Cytochrome c oxidase subunit 4 isoform 1, mitochondrial | 1.5093 | 1.6917 | 1.5931 | 1.7446 |  | 0.01397 | 0.02260 | 0.00215 | 0.00311 |
| CP-137 | sp\|Q01405\|SC23A | Protein transport protein Sec23A | 1.2349 | 1.3554 | 2.0614 | 1.5732 |  | 0.06259 | 0.10583 | 0.03918 | 0.08084 |
| CP-138 | sp\|P97807-2\|FUMH | Isoform Cytoplasmic of Fumarate hydratase, mitochondrial | 1.4707 | 1.4707 | 1.3684 | 1.3035 |  | 0.27572 | 0.27572 | 0.04603 | 0.16086 |
| CP-139 | sp\|Q920E5\|FPPS | Farnesyl pyrophosphate synthase | 1.8221 | 1.7824 | 1.2490 | 1.1164 |  | 0.00147 | 0.00403 | 0.02641 | 0.44784 |
| CP-140 | tr\|F7DBB3\|F7DBB3 | Protein Ahnak2 (Fragment) | 1.3134 | 1.4123 | 1.7275 | 1.6268 |  | 0.00920 | 0.01868 | 0.01782 | 0.02628 |
| CP-141 | sp\|P62281\|RS11 | 40S ribosomal protein S11 | 1.3729 | 1.1723 | 0.9985 | 0.8597 |  | 0.04029 | 0.05097 | 0.97714 | 0.15159 |
| CP-142 | sp\|P37889-2\|FBLN2 | Isoform 2 of Fibulin-2 | 0.5118 | 0.5684 | 0.6081 | 0.4975 |  | 0.00008 | 0.00025 | 0.00006 | 0.00002 |
| CP-143 | sp\|P10649\|GSTM1 | Glutathione S-transferase Mu 1 | 1.7439 | 1.7439 | 1.9360 | 1.5980 |  | 0.00565 | 0.00565 | 0.00086 | 0.00037 |
| CP-144 | sp\|P68433\|H31 | Histone H3.1 | 1.5551 | 1.4777 | 1.3561 | 1.1603 |  | 0.00020 | 0.00018 | 0.02243 | 0.06643 |
| CP-145 | sp\|Q9JII6\|AK1A1 | Alcohol dehydrogenase [NADP(+)] | 0.9860 | 0.9933 | 1.4670 | 1.4242 |  | 0.46904 | 0.45760 | 0.00963 | 0.00730 |
| CP-146 | tr\|E9Q1Z0\|E9Q1Z0 | Protein 4732456N10Rik | 1.7877 | 1.8726 | 3.9563 | 3.5767 |  | 0.07313 | 0.05058 | 0.00497 | 0.00426 |
| CP-147 | sp\|Q64378\|FKBP5 | Peptidyl-prolyl cis-trans isomerase FKBP5 | 2.0369 | 2.0745 | 2.1551 | 1.9691 |  | 0.04873 | 0.05009 | 0.01459 | 0.01474 |
| CP-148 | sp\|P43277\|H13 | Histone H1.3 | 2.4272 | 2.4272 | 1.7792 | 2.3341 |  | 0.15044 | 0.15044 | 0.04421 | 0.02330 |
| CP-149 | sp\|O35887\|CALU | Calumenin | 0.8334 | 1.4742 | 1.4594 | 1.1575 |  | 0.04739 | 0.01122 | 0.13698 | 0.07967 |
| CP-150 | sp\|P18761\|CAH6 | Carbonic anhydrase 6 | 1.8520 | 1.8520 | 1.7155 | 1.8792 |  | 0.27088 | 0.27088 | 0.03042 | 0.03537 |
| CP-151 | sp\|Q60930\|VDAC2 | Voltage-dependent anion-selective channel protein 2 | 1.5180 | 1.5374 | 1.8550 | 2.0491 |  | 0.01271 | 0.01069 | 0.01112 | 0.04418 |
| CP-152 | sp\|Q9D0F3\|LMAN1 | Protein ERGIC-53 | 1.4051 | 1.4051 | 1.4975 | 1.5417 |  | 0.09545 | 0.09545 | 0.02894 | 0.01459 |
| CP-153 | sp\|Q6P5H2-2\|NEST | Isoform 2 of Nestin | 0.7781 | 0.7749 | 0.6587 | 0.6912 |  | 0.02244 | 0.02128 | 0.00430 | 0.00459 |
| CP-154 | sp\|P09528\|FRIH | Ferritin heavy chain | 2.0058 | 2.0606 | 2.0005 | 1.4761 |  | 0.02813 | 0.00602 | 0.06417 | 0.06079 |
| CP-155 | sp\|P47753\|CAZA1 | F-actin-capping protein subunit alpha-1 | 1.6519 | 1.6519 | 1.1372 | 1.2321 |  | 0.04434 | 0.04434 | 0.00410 | 0.02311 |
| CP-156 | sp\|P62717\|RL18A | 60S ribosomal protein L18a | 1.3738 | 1.2176 | 1.3058 | 0.6035 |  | 0.01242 | 0.01690 | 0.10919 | 0.31974 |
| CP-157 | sp\|P62918\|RL8 | 60S ribosomal protein L8 | 2.1095 | 1.9299 | 1.7158 | 1.7687 |  | 0.00887 | 0.02500 | 0.00717 | 0.01432 |
| CP-158 | sp\|Q8K009\|AL1L2 | Mitochondrial 10-formyltetrahydrofolate dehydrogenase | 1.4425 | 1.4425 | 1.5975 | 1.4888 |  | 0.39846 | 0.39846 | 0.00324 | 0.04086 |
| CP-159 | sp\|Q8BJY1\|PSMD5 | 26S proteasome non-ATPase regulatory subunit 5 | 1.3084 | 1.2436 | 1.1316 | 1.1071 |  | 0.02619 | 0.07143 | 0.38432 | 0.76273 |
| CP-160 | sp\|P14069\|S10A6 | Protein S100-A6 | 0.6979 | 0.8596 | 0.8110 | 0.7747 |  | 0.00944 | 0.24955 | 0.04923 | 0.06209 |
| CP-161 | sp\|Q60931\|VDAC3 | Voltage-dependent anion-selective channel protein 3 | 1.3748 | 1.3748 | 1.5533 | 1.5285 |  | 0.05315 | 0.05315 | 0.01242 | 0.00984 |
| CP-162 | tr\|Q3U452\|Q3U452 | ATP synthase subunit alpha | 1.0691 | 1.3077 | 1.5640 | 1.6949 |  | 0.12265 | 0.09890 | 0.00535 | 0.00368 |
| CP-163 | sp\|Q8BH95\|ECHM | Enoyl-CoA hydratase, mitochondrial | 1.4303 | 1.4303 | 1.7722 | 1.5052 |  | 0.02659 | 0.02659 | 0.00073 | 0.00202 |
| CP-164 | sp\|Q3TW96\|UAP1L | UDP-N-acetylhexosamine pyrophosphorylase-like protein 1 | 1.1881 | 1.1881 | 1.6118 | 1.3916 |  | 0.45347 | 0.45347 | 0.00447 | 0.01750 |
| CP-165 | sp\|Q9R1P0\|PSA4 | Proteasome subunit alpha type-4 | 0.6298 | 0.6298 | 1.5719 | 0.8786 |  | 0.66174 | 0.66174 | 0.02382 | 0.03015 |
| CP-166 | sp\|Q6P5E4\|UGGG1 | UDP-glucose:glycoprotein glucosyltransferase 1 | 1.3508 | 1.3508 | 1.2070 | 1.1795 |  | 0.03149 | 0.03149 | 0.22745 | 0.04446 |
| CP-167 | sp\|P35486\|ODPA | Pyruvate dehydrogenase E1 component subunit alpha, somatic form, mitochondrial | 1.2380 | 1.2269 | 1.3249 | 1.3127 |  | 0.10615 | 0.10794 | 0.00896 | 0.02137 |
| CP-168 | sp\|P08074\|CBR2 | Carbonyl reductase [NADPH] 2 | 1.9692 | 1.9692 | 1.9712 | 1.8619 |  | 0.05287 | 0.05287 | 0.00221 | 0.01166 |
| CP-169 | sp\|P62204\|CALM | Calmodulin | 0.8061 | 0.9723 | 1.3636 | 1.1910 |  | 0.00014 | 0.00218 | 0.00287 | 0.00970 |
| CP-170 | sp\|O88844\|IDHC | Isocitrate dehydrogenase [NADP] cytoplasmic | 1.5302 | 2.4587 | 1.8365 | 2.4662 |  | 0.04299 | 0.02369 | 0.07468 | 0.03220 |
| CP-171 | tr\|A0PJE6\|A0PJE6 | Pccb protein (Fragment) | 0.9645 | 1.6108 | 1.9518 | 1.6949 |  | 0.23159 | 0.04328 | 0.05344 | 0.19834 |
| CP-172 | sp\|P35980\|RL18 | 60S ribosomal protein L18 | 2.2515 | 2.0700 | 1.6497 | 1.7836 |  | 0.00326 | 0.00491 | 0.00275 | 0.00039 |
| CP-173 | sp\|Q99MR6-2\|SRRT | Isoform B of Serrate RNA effector molecule homolog | 1.1467 | 1.2828 | 1.3793 | 1.1647 |  | 0.06223 | 0.00269 | 0.01783 | 0.03773 |
| CP-174 | sp\|Q8BP67\|RL24 | 60S ribosomal protein L24 | 1.7222 | 1.5191 | 1.3099 | 1.0702 |  | 0.00064 | 0.00393 | 0.32731 | 0.81867 |
| CP-175 | sp\|P37804\|TAGL | Transgelin | 0.5862 | 0.5620 | 0.4639 | 0.4488 |  | 0.00246 | 0.00075 | 0.00007 | 0.00019 |
| CP-176 | sp\|P70670\|NACAM | Nascent polypeptide-associated complex subunit alpha, muscle-specific form | 0.5723 | 0.5723 | 0.7431 | 0.6217 |  | 0.31298 | 0.31298 | 0.01193 | 0.04458 |
| CP-177 | sp\|Q8BJS4-2\|SUN2 | Isoform 2 of SUN domain-containing protein 2 | 1.5310 | 1.5747 | 1.2567 | 1.4593 |  | 0.02376 | 0.02602 | 0.07686 | 0.17079 |
| CP-178 | sp\|P47963\|RL13 | 60S ribosomal protein L13 | 1.7246 | 1.6035 | 1.3109 | 1.3396 |  | 0.00008 | 0.00007 | 0.00005 | 0.00001 |
| CP-179 | sp\|P62814\|VATB2 | V-type proton ATPase subunit B, brain isoform | 1.4278 | 1.4278 | 1.5588 | 1.3518 |  | 0.49415 | 0.49415 | 0.03581 | 0.17332 |
| CP-180 | sp\|P43276\|H15 | Histone H1.5 | 2.5763 | 2.5763 | 1.2343 | 1.9228 |  | 0.15352 | 0.15352 | 0.08966 | 0.03213 |
| CP-181 | sp\|P34022\|RANG | Ran-specific GTPase-activating protein | 0.8085 | 0.6908 | 0.7460 | 0.6984 |  | 0.16244 | 0.13558 | 0.05097 | 0.02958 |
| CP-182 | sp\|Q9WV27\|AT1A4 | Sodium/potassium-transporting ATPase subunit alpha-4 | 1.2150 | 1.2312 | 1.2299 | 1.3748 |  | 0.05964 | 0.03839 | 0.00671 | 0.00372 |
| CP-183 | sp\|Q8BL97-2\|SRSF7 | Isoform 2 of Serine/arginine-rich splicing factor 7 | 1.4465 | 1.5002 | 1.3860 | 1.1469 |  | 0.00660 | 0.00823 | 0.00340 | 0.00060 |
| CP-184 | sp\|Q9QYB1\|CLIC4 | Chloride intracellular channel protein 4 | 1.5961 | 1.6585 | 1.3894 | 1.5956 |  | 0.14919 | 0.21841 | 0.03568 | 0.14390 |
| CP-185 | sp\|Q60597-2\|ODO1 | Isoform 2 of 2-oxoglutarate dehydrogenase, mitochondrial | 0.9447 | 1.0167 | 1.4555 | 1.1469 |  | 0.18011 | 0.07528 | 0.00498 | 0.00743 |
| CP-186 | sp\|P56395\|CYB5 | Cytochrome b5 | 1.8275 | 1.8275 | 2.3045 | 1.9379 |  | 0.11904 | 0.11904 | 0.03486 | 0.02741 |
| CP-187 | sp\|Q8BZW2\|ANKRD56 | Ankyrin repeat domain-containing protein 56 | 1.8855 | 3.2434 | 3.2198 | 3.2934 |  | 0.01486 | 0.01486 | 0.01211 | 0.00946 |
| CP-188 | tr\|H9KV00\|H9KV00 | Protein SON | 0.7972 | 0.6556 | 1.0368 | 0.9007 |  | 0.15493 | 0.04398 | 0.92683 | 0.68815 |
| CP-189 | sp\|Q8BSY0\|ASPH | Aspartyl/asparaginyl beta-hydroxylase | 0.8884 | 1.0860 | 1.4506 | 1.4793 |  | 0.21480 | 0.14292 | 0.03184 | 0.16507 |
| CP-190 | sp\|P51859\|HDGF | Hepatoma-derived growth factor | 0.6892 | 0.6892 | 0.8235 | 0.7132 |  | 0.04120 | 0.04120 | 0.06320 | 0.02138 |
| CP-191 | sp\|Q9DB20\|ATPO | ATP synthase subunit O, mitochondrial | 1.1826 | 1.1826 | 1.4707 | 1.3029 |  | 0.13408 | 0.13408 | 0.01131 | 0.01837 |
| CP-192 | sp\|Q922Q8\|LRC59 | Leucine-rich repeat-containing protein 59 | 0.6786 | 0.7188 | 0.7980 | 0.7321 |  | 0.00006 | 0.00455 | 0.02351 | 0.00035 |
| CP-193 | sp\|Q8R010\|AIMP2 | Aminoacyl tRNA synthase complex-interacting multifunctional protein 2 | 1.1896 | 0.8721 | 1.2375 | 0.6406 |  | 0.29226 | 0.13063 | 0.76015 | 0.00641 |
| CP-194 | sp\|P01902\|HA1D | H-2 class I histocompatibility antigen, K-D alpha chain | 1.8994 | 1.8994 | 1.4873 | 1.5352 |  | 0.04604 | 0.04604 | 0.03902 | 0.03833 |
| CP-195 | tr\|E9Q6R7\|E9Q6R7 | Protein Utrn | 1.0991 | 1.3226 | 0.9644 | 1.0964 |  | 0.11905 | 0.03753 | 0.00233 | 0.02290 |
| CP-196 | sp\|Q60766-2\|IRGM1 | Isoform 2 of Immunity-related GTPase family M protein 1 | 1.0747 | 1.4920 | 2.1026 | 2.1837 |  | 0.09249 | 0.07315 | 0.07089 | 0.03165 |
| CP-197 | sp\|Q9D1R9\|RL34 | 60S ribosomal protein L34 | 1.4568 | 1.1776 | 0.9847 | 0.8319 |  | 0.01712 | 0.04718 | 0.83817 | 0.52495 |
| CP-198 | sp\|P00342\|LDHC | L-lactate dehydrogenase C chain | 1.9033 | 1.9033 | 1.5165 | 1.6779 |  | 0.47171 | 0.47171 | 0.04603 | 0.04724 |
| CP-199 | sp\|P62071\|RRAS2 | Ras-related protein R-Ras2 | 1.8341 | 1.8341 | 1.2599 | 1.5066 |  | 0.01449 | 0.01449 | 0.04344 | 0.01459 |
| CP-200 | sp\|Q9CW46\|RAVR1 | Ribonucleoprotein PTB-binding 1 | 1.0036 | 1.3726 | 0.9537 | 1.1703 |  | 0.46839 | 0.01908 | 0.88801 | 0.08992 |
| CP-201 | sp\|P51174\|ACADL | Long-chain specific acyl-CoA dehydrogenase, mitochondrial | 1.4596 | 1.6776 | 1.8814 | 1.8761 |  | 0.00726 | 0.01179 | 0.04350 | 0.12956 |
| CP-202 | sp\|Q11011\|PSA | Puromycin-sensitive aminopeptidase | 1.2597 | 1.3019 | 1.2083 | 1.2348 |  | 0.04426 | 0.01539 | 0.00682 | 0.05814 |
| CP-203 | sp\|Q9CY58-2\|PAIRB | Isoform 2 of Plasminogen activator inhibitor 1 RNA-binding protein | 0.6910 | 0.6569 | 0.6591 | 0.5060 |  | 0.05159 | 0.02231 | 0.20456 | 0.06263 |
| CP-204 | tr\|E9PYB0\|E9PYB0 | Protein Ahnak2 (Fragment) | 1.3974 | 1.4522 | 1.7211 | 1.6177 |  | 0.00880 | 0.01002 | 0.04429 | 0.01817 |
| CP-205 | sp\|Q05816\|FABP5 | Fatty acid-binding protein, epidermal | 1.1469 | 1.2477 | 1.5487 | 1.3907 |  | 0.20309 | 0.03776 | 0.00559 | 0.00482 |
| CP-206 | sp\|Q05186\|RCN1 | Reticulocalbin-1 | 1.0670 | 1.1440 | 1.5569 | 1.4679 |  | 0.02740 | 0.13884 | 0.05228 | 0.04049 |
| CP-207 | sp\|Q02788\|CO6A2 | Collagen alpha-2(VI) chain | 1.9584 | 1.9584 | 2.1479 | 2.1606 |  | 0.01473 | 0.01473 | 0.00377 | 0.00121 |
| CP-208 | sp\|Q9DCX2\|ATP5H | ATP synthase subunit d, mitochondrial | 1.3291 | 1.3291 | 1.4908 | 1.4464 |  | 0.13442 | 0.13442 | 0.00569 | 0.00585 |
| CP-209 | sp\|P46664\|PURA2 | Adenylosuccinate synthetase isozyme 2 | 0.4928 | 0.4928 | 0.5011 | 0.5850 |  | 0.49082 | 0.49082 | 0.01144 | 0.01823 |
| CP-210 | tr\|E9QAZ2\|E9QAZ2 | Ribosomal protein L15 | 1.4981 | 1.4252 | 1.1754 | 1.2129 |  | 0.00349 | 0.00332 | 0.22803 | 0.01490 |
| CP-211 | sp\|P17710-2\|HXK1 | Isoform HK1-SB of Hexokinase-1 | 1.2157 | 1.4097 | 2.2756 | 1.8805 |  | 0.11425 | 0.07691 | 0.02317 | 0.01733 |
| CP-212 | sp\|P11440\|CDK1 | Cyclin-dependent kinase 1 | 0.7321 | 0.7321 | 0.6431 | 0.7009 |  | 0.24810 | 0.24810 | 0.01465 | 0.01843 |
| CP-213 | tr\|Q6PJ91\|Q6PJ91 | Gstm7 protein | 1.6444 | 1.6444 | 1.8723 | 1.7245 |  | 0.14786 | 0.14786 | 0.00515 | 0.01360 |
| CP-214 | sp\|P41731\|CD63 | CD63 antigen | 1.1867 | 1.1867 | 1.3672 | 1.4033 |  | 0.03356 | 0.03356 | 0.20928 | 0.02364 |
| CP-215 | tr\|D3Z158\|D3Z158 | Protein Qars | 1.1415 | 1.4353 | 1.3567 | 1.3057 |  | 0.16221 | 0.07765 | 0.00759 | 0.09745 |
| CP-216 | sp\|P62821\|RAB1A | Ras-related protein Rab-1A | 1.2178 | 1.2178 | 1.3212 | 1.2988 |  | 0.00083 | 0.00083 | 0.01754 | 0.00061 |
| CP-217 | sp\|Q91VW3\|SH3L3 | SH3 domain-binding glutamic acid-rich-like protein 3 | 0.8166 | 1.2375 | 1.8471 | 1.8692 |  | 0.07066 | 0.00888 | 0.01195 | 0.01330 |
| CP-218 | sp\|P12970\|RL7A | 60S ribosomal protein L7a | 1.2612 | 1.2665 | 1.3224 | 1.3178 |  | 0.08397 | 0.07958 | 0.00478 | 0.08655 |
| CP-219 | sp\|P31786\|ACBP | Acyl-CoA-binding protein | 1.4522 | 1.4522 | 2.0350 | 1.6086 |  | 0.20580 | 0.20580 | 0.00343 | 0.02666 |
| CP-220 | sp\|P61255\|RL26 | 60S ribosomal protein L26 | 1.5028 | 1.3869 | 1.2223 | 1.2029 |  | 0.00010 | 0.00020 | 0.04972 | 0.04893 |
| CP-221 | sp\|P32020-2\|NLTP | Isoform SCP2 of Non-specific lipid-transfer protein | 1.2053 | 1.2053 | 1.4107 | 1.2510 |  | 0.33393 | 0.33393 | 0.02308 | 0.16845 |
| CP-222 | sp\|P45952\|ACADM | Medium-chain specific acyl-CoA dehydrogenase, mitochondrial | 1.1360 | 1.1360 | 1.3152 | 1.2436 |  | 0.02727 | 0.02727 | 0.00721 | 0.00316 |
| CP-223 | sp\|P00405\|COX2 | Cytochrome c oxidase subunit 2 | 1.0975 | 1.0975 | 1.3861 | 1.6262 |  | 0.24319 | 0.24312 | 0.11881 | 0.03510 |
| CP-224 | sp\|P50544\|ACADV | Very long-chain specific acyl-CoA dehydrogenase, mitochondrial | 1.1228 | 1.1228 | 1.4131 | 1.1003 |  | 0.39089 | 0.39089 | 0.03988 | 0.25846 |
| CP-225 | sp\|Q9EPU0-2\|RENT1 | Isoform 2 of Regulator of nonsense transcripts 1 | 0.9798 | 0.9798 | 0.4878 | 0.7816 |  | 0.91269 | 0.91269 | 0.04264 | 0.09019 |
| CP-226 | sp\|Q07417\|ACADS | Short-chain specific acyl-CoA dehydrogenase, mitochondrial | 1.3844 | 1.3844 | 1.6551 | 1.5009 |  | 0.14048 | 0.14048 | 0.00366 | 0.01228 |
| CP-227 | sp\|P30416\|FKBP4 | Peptidyl-prolyl cis-trans isomerase FKBP4 | 0.8276 | 0.8276 | 0.6972 | 0.7570 |  | 0.39987 | 0.39987 | 0.00734 | 0.07930 |
| CP-228 | sp\|Q921M3-2\|SF3B3 | Isoform 2 of Splicing factor 3B subunit 3 | 1.4372 | 1.2375 | 1.1187 | 0.9809 |  | 0.01013 | 0.16417 | 0.79004 | 0.95093 |
| CP-229 | sp\|Q9WUA2\|SYFB | Phenylalanine--tRNA ligase beta subunit | 1.2172 | 1.1562 | 1.4252 | 1.1534 |  | 0.10103 | 0.16817 | 0.04332 | 0.04397 |
| CP-230 | sp\|Q62426\|CYTB | Cystatin-B | 1.2025 | 1.2664 | 1.6881 | 1.7599 |  | 0.02179 | 0.07658 | 0.02216 | 0.04316 |
| CP-231 | sp\|Q99LT0\|DPY30 | Protein dpy-30 homolog | 0.6031 | 0.7099 | 0.8046 | 0.8573 |  | 0.00713 | 0.00695 | 0.50952 | 0.21828 |
| CP-232 | sp\|Q9CZ42-2\|NNRD | Isoform 2 of ATP-dependent (S)-NAD(P)H-hydrate dehydratase | 1.1939 | 1.1939 | 1.4713 | 1.4159 |  | 0.24089 | 0.24089 | 0.02809 | 0.13511 |
| CP-233 | sp\|P84104-2\|SRSF3 | Isoform Short of Serine/arginine-rich splicing factor 3 | 1.3138 | 1.3648 | 1.3069 | 1.1964 |  | 0.01816 | 0.02180 | 0.06893 | 0.04313 |
| CP-234 | sp\|Q61576\|FKB10 | Peptidyl-prolyl cis-trans isomerase FKBP10 | 1.3711 | 1.3711 | 1.1077 | 1.2440 |  | 0.01284 | 0.01284 | 0.12685 | 0.00427 |
| CP-235 | sp\|P05201\|AATC | Aspartate aminotransferase, cytoplasmic | 1.7704 | 1.7704 | 1.3689 | 1.3967 |  | 0.21191 | 0.21191 | 0.02949 | 0.18506 |
| CP-236 | sp\|Q8CJ53-3\|CIP4 | Isoform 3 of Cdc42-interacting protein 4 | 0.9150 | 1.3085 | 1.0119 | 1.1747 |  | 0.22620 | 0.03674 | 0.79507 | 0.23861 |
| CP-237 | sp\|Q9DBS1\|TMM43 | Transmembrane protein 43 | 1.4113 | 1.4113 | 1.2234 | 1.3819 |  | 0.41245 | 0.41245 | 0.05360 | 0.03267 |
| CP-238 | sp\|Q99JT2\|MST4 | Serine/threonine-protein kinase MST4 | 1.0782 | 1.0782 | 1.3146 | 1.2165 |  | 0.47442 | 0.47442 | 0.04032 | 0.06117 |
| CP-239 | sp\|Q9DBP5\|KCY | UMP-CMP kinase | 0.9730 | 0.9730 | 1.5635 | 1.1664 |  | 0.76404 | 0.76404 | 0.01821 | 0.22710 |
| CP-240 | sp\|Q9WTR5\|CAD13 | Cadherin-13 | 2.0954 | 2.0954 | 1.8591 | 1.8369 |  | 0.25489 | 0.25489 | 0.04402 | 0.04304 |
| CP-241 | sp\|Q9WTN3-2\|SRBP1 | Isoform SREBP-1A-W42 of Sterol regulatory element-binding protein 1 | 1.4946 | 1.3585 | 1.9482 | 1.6094 |  | 0.02733 | 0.05706 | 0.19541 | 0.08604 |
| CP-242 | sp\|P19137\|LAMA1 | Laminin subunit alpha-1 | 1.3903 | 1.3334 | 2.3743 | 1.9097 |  | 0.08888 | 0.09214 | 0.03610 | 0.02127 |
| CP-243 | sp\|Q6ZWQ0\|SYNE2 | Nesprin-2 | 1.1459 | 1.2583 | 1.4938 | 1.6404 |  | 0.07464 | 0.01889 | 0.00689 | 0.03393 |
| CP-244 | sp\|Q8K1R3\|PNPT1 | Polyribonucleotide nucleotidyltransferase 1, mitochondrial | 0.8393 | 1.7021 | 1.3951 | 1.5930 |  | 0.10775 | 0.01959 | 0.15812 | 0.18816 |
| CP-245 | sp\|Q9CQM9\|GLRX3 | Glutaredoxin-3 | 1.0404 | 1.1933 | 1.8648 | 1.4987 |  | 0.11499 | 0.18970 | 0.38479 | 0.00077 |
| CP-246 | tr\|B2RY51\|B2RY51 | Thyroid hormone receptor interactor 11 | 1.6133 | 1.4831 | 1.7080 | 1.2816 |  | 0.14373 | 0.06121 | 0.01593 | 0.55379 |
| CP-247 | sp\|Q9ESX5\|DKC1 | H/ACA ribonucleoprotein complex subunit 4 | 0.6037 | 0.6037 | 0.9544 | 0.8625 |  | 0.03627 | 0.03627 | 0.15903 | 0.03237 |
| CP-248 | sp\|Q9WU28\|PFD5 | Prefoldin subunit 5 | 0.8022 | 0.8022 | 1.4259 | 1.2356 |  | 0.70473 | 0.70473 | 0.00089 | 0.03224 |
| CP-249 | sp\|A3KGK3-2\|FR1L4 | Isoform 2 of Fer-1-like protein 4 | 1.1123 | 1.1698 | 1.5243 | 1.2516 |  | 0.15111 | 0.16175 | 0.04967 | 0.07753 |
| CP-250 | sp\|Q91YR9\|PTGR1 | Prostaglandin reductase 1 | 1.3397 | 1.5072 | 1.3322 | 1.6618 |  | 0.00084 | 0.00547 | 0.03753 | 0.00191 |
| CP-251 | sp\|Q7TMY8-2\|HUWE1 | Isoform 2 of E3 ubiquitin-protein ligase HUWE1 | 0.7715 | 0.6568 | 0.7040 | 0.6879 |  | 0.12346 | 0.00151 | 0.08551 | 0.13216 |
| CP-252 | sp\|Q8CCF0-2\|PRP31 | Isoform 2 of U4/U6 small nuclear ribonucleoprotein Prp31 | 1.4644 | 1.3542 | 1.4677 | 1.2695 |  | 0.03116 | 0.33446 | 0.59199 | 0.37635 |
| CP-253 | sp\|Q8BL74\|TF3C2 | General transcription factor 3C polypeptide 2 | 0.6871 | 0.6871 | 0.4203 | 0.6398 |  | 0.62126 | 0.62126 | 0.04036 | 0.24608 |
| CP-254 | tr\|Q5PRE9\|Q5PRE9 | Ncor1 protein | 1.6893 | 1.6893 | 1.2339 | 1.3554 |  | 0.02514 | 0.02514 | 0.02510 | 0.03372 |
| CP-255 | sp\|P11688\|ITA5 | Integrin alpha-5 | 1.7362 | 1.6228 | 1.2212 | 1.1084 |  | 0.00072 | 0.00034 | 0.04131 | 0.29067 |
| CP-256 | tr\|Q8BVP2\|Q8BVP2 | L-lactate dehydrogenase | 1.3649 | 1.5517 | 2.0909 | 2.9994 |  | 0.05258 | 0.01839 | 0.38926 | 0.39106 |
| CP-257 | sp\|P24288\|BCAT1 | Branched-chain-amino-acid aminotransferase, cytosolic | 0.7115 | 0.7115 | 0.5692 | 0.6288 |  | 0.00138 | 0.00138 | 0.00016 | 0.00004 |
| CP-258 | sp\|Q9EQW8\|NDST4 | Bifunctional heparan sulfate N-deacetylase/N-sulfotransferase 4 | 1.4852 | 1.5241 | 1.2490 | 1.4602 |  | 0.04407 | 0.01289 | 0.03909 | 0.00476 |
| CP-259 | sp\|Q62093\|SRSF2 | Serine/arginine-rich splicing factor 2 | 1.2907 | 1.2320 | 1.4027 | 1.1090 |  | 0.03687 | 0.05239 | 0.03589 | 0.20961 |
| CP-260 | sp\|Q99MZ6\|MYO7B | Unconventional myosin-VIIb | 2.3779 | 2.3779 | 3.8795 | 2.9644 |  | 0.08108 | 0.08108 | 0.00748 | 0.00866 |
| CP-261 | sp\|Q921M4-2\|GOGA2 | Isoform 2 of Golgin subfamily A member 2 | 1.2982 | 1.4023 | 1.8029 | 1.7010 |  | 0.14907 | 0.08082 | 0.03330 | 0.05185 |
| CP-262 | sp\|Q45VK7-2\|DYHC2 | Isoform 2 of Cytoplasmic dynein 2 heavy chain 1 | 1.4320 | 2.2747 | 1.2732 | 1.6785 |  | 0.03365 | 0.03766 | 0.01450 | 0.28661 |
| CP-263 | sp\|Q5DTN8\|JKIP3 | Janus kinase and microtubule-interacting protein 3 | 0.7826 | 0.7290 | 0.7532 | 0.6086 |  | 0.02378 | 0.00569 | 0.00287 | 0.00401 |
| CP-264 | sp\|Q9QY76\|VAPB | Vesicle-associated membrane protein-associated protein B | 1.3618 | 1.1398 | 1.2036 | 1.1999 |  | 0.00221 | 0.06596 | 0.44143 | 0.13807 |
| CP-265 | tr\|Q9CWC9\|Q9CWC9 | Putative uncharacterized protein | 1.6117 | 1.6117 | 2.1756 | 1.6279 |  | 0.11044 | 0.11044 | 0.01816 | 0.01360 |
| CP-266 | sp\|Q99PF4-2\|CAD23 | Isoform 2 of Cadherin-23 | 1.8090 | 1.8090 | 1.3493 | 1.4940 |  | 0.07454 | 0.07454 | 0.00259 | 0.03789 |
| CP-267 | sp\|Q5D525-3\|SYC1L | Isoform 3 of Synaptonemal complex central element protein 1-like | 1.6872 | 1.6832 | 1.1932 | 1.1071 |  | 0.01927 | 0.01457 | 0.50487 | 0.12051 |
| CP-268 | tr\|F8VPY2\|F8VPY2 | Transcription initiation factor TFIID subunit 5 | 1.2883 | 1.4619 | 1.2315 | 1.3103 |  | 0.04031 | 0.04684 | 0.14489 | 0.07708 |
| CP-269 | sp\|E9PZQ0\|RYR1 | Ryanodine receptor 1 | 0.5475 | 0.6301 | 0.9493 | 0.8722 |  | 0.01770 | 0.05923 | 0.77519 | 0.28681 |
| CP-270 | sp\|Q5S006\|LRRK2 | Leucine-rich repeat serine/threonine-protein kinase 2 | 1.3416 | 1.6597 | 3.4772 | 4.1396 |  | 0.02161 | 0.10699 | 0.30339 | 0.35659 |
| CP-271 | tr\|B2RWW2\|B2RWW2 | Golgb1 protein | 1.5045 | 1.4480 | 1.2983 | 1.5871 |  | 0.10985 | 0.09294 | 0.00348 | 0.01932 |
| CP-272 | sp\|Q80XB4-2\|NRAP | Isoform 2 of Nebulin-related-anchoring protein | 0.7448 | 0.7448 | 0.6627 | 0.5225 |  | 0.53961 | 0.53961 | 0.07109 | 0.04388 |
| CP-273 | sp\|Q8CIS0-2\|CAR11 | Isoform 2 of Caspase recruitment domain-containing protein 11 | 1.3808 | 1.3112 | 1.6072 | 1.8809 |  | 0.02409 | 0.11476 | 0.48271 | 0.25091 |
| CP-274 | sp\|Q8K2I9-2\|FBX18 | Isoform 2 of F-box only protein 18 | 0.7653 | 0.6206 | 0.5271 | 0.5363 |  | 0.00946 | 0.01070 | 0.04086 | 0.05459 |
| CP-275 | sp\|Q922S8\|KIF2C | Kinesin-like protein KIF2C | 1.3338 | 1.2994 | 1.1884 | 1.1585 |  | 0.00507 | 0.00190 | 0.18412 | 0.04796 |
| CP-276 | sp\|Q9CQA3\|DHSB | Succinate dehydrogenase [ubiquinone] iron-sulfur subunit, mitochondrial | 1.0314 | 1.0314 | 1.5713 | 1.3528 |  | 0.85526 | 0.85526 | 0.00781 | 0.12696 |
| CP-277 | sp\|A2AAJ9-2\|OBSCN | Isoform 2 of Obscurin | 1.4929 | 2.0516 | 2.0766 | 2.1998 |  | 0.11450 | 0.01633 | 0.44539 | 0.18867 |
| CP-278 | sp\|A1L3T7\|FA65C | Protein FAM65C | 0.8421 | 0.9024 | 0.7766 | 0.6656 |  | 0.18633 | 0.26047 | 0.00343 | 0.03985 |
| CP-279 | sp\|P07146\|TRY2 | Anionic trypsin-2 | 3.1895 | 3.1895 | 4.2936 | 3.2034 |  | 0.02109 | 0.02109 | 0.01422 | 0.00945 |
| CP-280 | tr\|G0XVH0\|G0XVH0 | Putative uncharacterized protein | 1.8077 | 1.8077 | 1.7336 | 1.7336 |  | 0.16623 | 0.16623 | 0.04293 | 0.11588 |

All differentially expressed proteins were listed following our analysis in FigS1. Protein ID, CP for Cha & Park; Accession Number, uniprot data base access number; Annotation, full names of the identified proteins; Average fold change, average values from 3 independent batches

**Table B. List of proteins in Fig 4A categorized into six groups based on their expression patterns during co-culture**

|  | **ProteinID** | **Gene Name** | **Gene Abbrev.** | **GenBank ID** |
| --- | --- | --- | --- | --- |
| **Group 1** | CP-20 | Beta-globin | A8DUK4 | tr\|A8DUK4\| |
|  | CP-27 | 60S ribosomal protein L4 | RL4 | sp\|Q9D8E6\| |
|  | CP-28 | Isoform HSP105-beta of Heat shock protein 105 kDa | HS105 | sp\|Q61699-2\| |
|  | CP-39 | Histone H4 | H4 | sp\|P62806\| |
|  | CP-48 | Serum albumin | ALBU | sp\|P07724\| |
|  | CP-50 | 60S ribosomal protein L3 | RL3 | sp\|P27659\| |
|  | CP-72 | Translationally-controlled tumor protein | TCTP | sp\|P63028\| |
|  | CP-104 | Sterol-4-alpha-carboxylate 3-dehydrogenase, decarboxylating | NSDHL | sp\|Q9R1J0\| |
|  | CP-109 | 40S ribosomal protein S9 | RS9 | sp\|Q6ZWN5\| |
|  | CP-117 | 60S ribosomal protein L7 | RL7 | sp\|P14148\| |
|  | CP-119 | CCA tRNA nucleotidyltransferase 1, mitochondrial | TRNT1 | sp\|Q8K1J6\| |
|  | CP-121 | 40S ribosomal protein S8 | RS8 | sp\|P62242\| |
|  | CP-128 | Putative uncharacterized protein | Q8C622 | tr\|Q8C622\| |
|  | CP-131 | 60S ribosomal protein L13a | RL13A | sp\|P19253\| |
|  | CP-141 | 40S ribosomal protein S11 | RS11 | sp\|P62281\| |
|  | CP-156 | 60S ribosomal protein L18a | RL18A | sp\|P62717\| |
|  | CP-157 | 60S ribosomal protein L8 | RL8 | sp\|P62918\| |
|  | CP-159 | 26S proteasome non-ATPase regulatory subunit 5 | PSMD5 | sp\|Q8BJY1\| |
|  | CP-166 | UDP-glucose:glycoprotein glucosyltransferase 1 | UGGG1 | sp\|Q6P5E4\| |
|  | CP-172 | 60S ribosomal protein L18 | RL18 | sp\|P35980\| |
|  | CP-178 | 60S ribosomal protein L13 | RL13 | sp\|P47963\| |
|  | CP-194 | H-2 class I histocompatibility antigen, K-D alpha chain | HA1D | sp\|P01902\| |
|  | CP-197 | 60S ribosomal protein L34 | RL34 | sp\|Q9D1R9\| |
|  | CP-199 | Ras-related protein R-Ras2 | RRAS2 | sp\|P62071\| |
|  | CP-212 | Cyclin-dependent kinase | CDK1 | sp\|P11440\| |
|  | CP-220 | 60S ribosomal protein L26 | RL26 | sp\|P61255\| |
|  | CP-225 | Isoform 2 of Regulator of nonsense transcripts 1 | RENT1 | sp\|Q9EPU0-2\| |
|  | CP-227 | Peptidyl-prolyl cis-trans isomerase FKBP4 | FKBP4 | sp\|P30416\| |
|  | CP-228 | Isoform 2 of Splicing factor 3B subunit 3 | SF3B3 | sp\|Q921M3-2\| |
|  | CP-233 | Isoform Short of Serine/arginine-rich splicing factor 3 | SRSF3 | sp\|P84104-2\| |
|  | CP-234 | Peptidyl-prolyl cis-trans isomerase FKBP10 | FKB10 | sp\|Q61576\| |
|  | CP-241 | Isoform SREBP-1A-W42 of Sterol regulatory element-binding protein 1 | SRBP1 | sp\|Q9WTN3-2\| |
|  | CP-252 | Isoform 2 of U4/U6 small nuclear ribonucleoprotein Prp31 | PRP31 | sp\|Q8CCF0-2\| |
|  | CP-253 | General transcription factor 3C polypeptide 2 | TF3C2 | sp\|Q8BL74\| |
|  | CP-254 | Ncor1 protein | Q5PRE9 | tr\|Q5PRE9\| |
|  | CP-258 | Bifunctional heparan sulfate N-deacetylase/N-sulfotransferase 4 | NDST4 | sp\|Q9EQW8\| |
|  | CP-264 | Vesicle-associated membrane protein-associated protein B | VAPB | sp\|Q9QY76\| |
|  | CP-270 | Leucine-rich repeat serine/threonine-protein kinase 2 | LRRK2 | sp\|Q5S006\| |
|  | CP-273 | Isoform 2 of Caspase recruitment domain-containing protein 11 | CAR11 | sp\|Q8CIS0-2\| |
|  | CP-275 | Kinesin-like protein KIF2C | KIF2C | sp\|Q922S8\| |
|  | **Protein ID** | **Gene Name** | **Gene Abbrev.** | **GenBank ID** |
| **Group 2** | CP-10 | L-lactate dehydrogenase A chain | LDHA | sp\|P06151\| |
|  | CP-11 | Clathrin heavy chain 1 | CLH1 | sp\|Q68FD5\| |
|  | CP-19 | Annexin A4 | ANXA4 | sp\|P97429\| |
|  | CP-21 | Isoform 2 of Filamin-C | FLNC | sp\|Q8VHX6-2\| |
|  | CP-37 | Protein transport protein Sec31A | SC31A | sp\|Q3UPL0\| |
|  | CP-70 | Ras GTPase-activating protein-binding protein 1 | G3BP1 | sp\|P97855\| |
|  | CP-77 | Coatomer subunit gamma-1 | COPG1 | sp\|Q9QZE5\| |
|  | CP-84 | Niban-like protein 1 | NIBL1 | sp\|Q8R1F1\| |
|  | CP-90 | Putative uncharacterized protein | Q3UZH2 | tr\|Q3UZH2\| |
|  | CP-99 | Intergrin beta-1 | IMA2 | sp\|P52293\| |
|  | CP-116 | Nucleoprotein TPR | TPR | sp\|F6ZDS4\| |
|  | CP-120 | Isocitrate dehydrogenase [NAD] subunit alpha, mitochondrial | IDH3A | sp\|Q9D6R2\| |
|  | CP-133 | Procollagen-lysine,2-oxoglutarate 5-dioxygenase 1 | PLOD1 | sp\|Q9R0E2\| |
|  | CP-144 | Histone H3.1 | H31 | sp\|P68433\| |
|  | CP-149 | Calumenin | CALU | sp\|O35887\| |
|  | CP-171 | Pccb protein (Fragment) | A0PJE6 | tr\|A0PJE6\| |
|  | CP-176 | Nascent polypeptide-associated complex subunit alpha | NACAM | sp\|P70670\| |
|  | CP-181 | Ran-specific GTPase-activating protein | RANG | sp\|P34022\| |
|  | CP-183 | Isoform 2 of Serine/arginine-rich splicing factor 7 | SRSF7 | sp\|Q8BL97-2\| |
|  | CP-193 | Aminoacyl tRNA synthase complex-interacting multifunctional protein 2 | AIMP2 | sp\|Q8R010\| |
|  | CP-195 | Protein Utrn | E9Q6R7 | tr\|E9Q6R7\| |
|  | CP-200 | Ribonucleoprotein PTB-binding 1 | RAVR1 | sp\|Q9CW46\| |
|  | CP-201 | Long-chain specific acyl-CoA dehydrogenase, mitochondrial | ACADL | sp\|P51174\| |
|  | CP-202 | Puromycin-sensitive aminopeptidase | PSA | sp\|Q11011\| |
|  | CP-236 | Isoform 3 of Cdc42-interacting protein 4 | CIP4 | sp\|Q8CJ53-3\| |
|  | CP-244 | Polyribonucleotide nucleotidyltransferase 1, mitochondrial | PNPT1 | sp\|Q8K1R3\| |
|  | CP-256 | L-lactate dehydrogenase | Q8BVP2 | tr\|Q8BVP2\| |
|  | CP-263 | Janus kinase and microtubule-interacting protein 3 | JKIP3 | sp\|Q5DTN8\| |
|  | CP-268 | Transcription initiation factor TFIID subunit 5 | F8VPY2 | tr\|F8VPY2\| |
|  | CP-272 | Isoform 2 of Nebulin-related-anchoring protein | NRAP | sp\|Q80XB4-2\| |
|  | CP-277 | Isoform 2 of Obscurin | OBSCN | sp\|A2AAJ9-2\| |
|  | **Protein ID** | **Gene Name** | **Gene Abbrev.** | **GenBank ID** |
| **Group 3** | CP-24 | Isoform 2 of Tropomyosin alpha-1 chain | TPM1 | sp\|P58771-2\| |
|  | CP-31 | Tropomyosin 1, alpha, isoform CRA_j | G5E8R1 | tr\|G5E8R1\| |
|  | CP-44 | Fascin | FSCN1 | sp\|Q61553\| |
|  | CP-56 | Actin (Fragment) | H9BUF4 | tr\|H9BUF4\| |
|  | CP-76 | Putative uncharacterized protein | Q9CY06 | tr\|Q9CY06\| |
|  | CP-79 | Putative uncharacterized protein (Fragment) | Q3U505 | tr\|Q3U505\| |
|  | CP-92 | 60S ribosomal protein L6 | RL6 | sp\|P47911\| |
|  | CP-106 | Cytochrome b-c1 complex subunit 2, mitochondrial | QCR2 | sp\|Q9DB77\| |
|  | CP-126 | Importin-7 | IPO7 | sp\|Q9EPL8\| |
|  | CP-139 | Farnesyl pyrophosphate synthase | FPPS | sp\|Q920E5\| |
|  | CP-153 | Isoform 2 of Nestin | NEST | sp\|Q6P5H2-2\| |
|  | CP-154 | Ferritin heavy chain | FRIH | sp\|P09528\| |
|  | CP-155 | F-actin-capping protein subunit alpha-1 | CAZA1 | sp\|P47753\| |
|  | CP-174 | 60S ribosomal protein L24 | RL24 | sp\|Q8BP67\| |
|  | CP-177 | Isoform 2 of SUN domain-containing protein 2 | SUN2 | sp\|Q8BJS4-2\| |
|  | CP-207 | Collagen alpha-1(VI) chain | CO6A1 | sp\|Q04857\| |
|  | CP-209 | Adenylosuccinate synthetase isozyme 2 | PURA2 | sp\|P46664\| |
|  | CP-210 | Ribosomal protein L15 | E9QAZ2 | tr\|E9QAZ2\| |
|  | CP-255 | Integrin alpha-5 | ITA5 | sp\|P11688\| |
|  | CP-257 | Branched-chain-amino-acid aminotransferase, cytosolic | BCAT1 | sp\|P24288\| |
|  | CP-262 | Isoform 2 of Cytoplasmic dynein 2 heavy chain 1 | DYHC2 | sp\|Q45VK7-2\| |
|  | CP-267 | Isoform 3 of Synaptonemal complex central element protein 1-like | SYC1L | sp\|Q5D525-3\| |
|  | **Protein ID** | **Gene Name** | **Gene Abbrev.** | **GenBank ID** |
| **Group 4** | CP-40 | Heterogeneous nuclear ribonucleoprotein H2 | HNRH2 | sp\|P70333\| |
|  | CP-103 | Importin subunit alpha-2 | ITB1 | sp\|P09055\| |
|  | CP-110 | Galectin-3-binding protein | LG3BP | sp\|Q07797\| |
|  | CP-111 | Krt6b protein (Fragment) | Q05B38 | tr\|Q05B38\| |
|  | CP-145 | Alcohol dehydrogenase [NADP(+)] | AK1A1 | sp\|Q9JII6\| |
|  | CP-168 | Carbonyl reductase [NADPH] 2 | CBR2 | sp\|P08074\| |
|  | CP-173 | Isoform B of Serrate RNA effector molecule homolog | SRRT | sp\|Q99MR6-2\| |
|  | CP-186 | Cytochrome b5 | CYB5 | sp\|P56395\| |
|  | CP-219 | Acyl-CoA-binding protein | ACBP | sp\|P31786\| |
|  | CP-242 | Laminin subunit alpha-1 | LAMA1 | sp\|P19137\| |
|  | CP-248 | Prefoldin subunit 5 | PFD5 | sp\|Q9WU28\| |
|  | CP-259 | Serine/arginine-rich splicing factor 2 | SRSF2 | sp\|Q62093\| |
|  | CP-260 | Unconventional myosin-VIIb | MYO7B | sp\|Q99MZ6\| |
|  | CP-265 | Putative uncharacterized protein | Q9CWC9 | tr\|Q9CWC9\| |
|  | **Protein ID** | **Gene Name** | **Gene Abbrev.** | **GenBank ID** |
| **Group 5** | CP-5 | Fructose-bisphosphate aldolase A | ALDOA | sp\|P05064\| |
|  | CP-51 | Sodium/potassium-transporting ATPase subunit alpha-1 | AT1A1 | sp\|Q8VDN2\| |
|  | CP-86 | 6-phosphogluconate dehydrogenase, decarboxylating | 6PGD | sp\|Q9DCD0\| |
|  | CP-114 | Succinyl-CoA:3-ketoacid coenzyme A transferase 1, mitochondrial | SCOT1 | sp\|Q9D0K2\| |
|  | CP-118 | Isoform LAMP-2B of Lysosome-associated membrane glycoprotein 2 | LAMP2 | sp\|P17047-2\| |
|  | CP-124 | Sodium/potassium-transporting ATPase subunit alpha-2 | AT1A2 | sp\|Q6PIE5\| |
|  | CP-127 | Glyceraldehyde-3-phosphate dehydrogenase | F6UT49 | tr\|F6UT49\| |
|  | CP-134 | Cytochrome c oxidase subunit 6B1 | CX6B1 | sp\|P56391\| |
|  | CP-180 | Histone H1.5 | H15 | sp\|P43276\| |
|  | CP-182 | Sodium/potassium-transporting ATPase subunit alpha-4 | AT1A4 | sp\|Q9WV27\| |
|  | CP-196 | Isoform 2 of Immunity-related GTPase family M protein 1 | IRGM1 | sp\|Q60766-2\| |
|  | CP-206 | Reticulocalbin-1 | RCN1 | sp\|Q05186\| |
|  | CP-214 | CD63 antigen | CD63 | sp\|P41731\| |
|  | CP-223 | Cytochrome c oxidase subunit 2 | COX2 | sp\|P00405\| |
|  | CP-237 | Transmembrane protein 43 | TMM43 | sp\|Q9DBS1\| |
|  | CP-245 | Glutaredoxin-3 | GLRX3 | sp\|Q9CQM9\| |
|  | CP-250 | Prostaglandin reductase 1 | PTGR1 | sp\|Q91YR9\| |
|  | CP-271 | Golgb1 protein | B2RWW2 | tr\|B2RWW2\| |
|  | **Protein ID** | **Gene Name** | **Gene Abbrev.** | **GenBank ID** |
| **Group 6** | CP-2 | SWI/SNF-related matrix-associated actin-dependent regulator of chromatin subfamily E member 1-HMG20B | HMGP20B | sp\|Q9Z104\| |
|  | CP-8 | Transcription factor E2-alpha | TCF3 | sp\|P15806\| |
|  | CP-61 | Catalase | Q91X12 | tr\|Q91XI2\| |
|  | CP-64 | Catalase | CATA | sp\|P24270\| |
|  | CP-94 | Dihydrolipoyl dehydrogenase, mitochondrial | DLDH | sp\|O08749\| |
|  | CP-107 | Costars family protein ABRACL | ABRAL | sp\|Q4KML4\| |
|  | CP-113 | Nucleobindin-2 | NUCB2 | sp\|P81117\| |
|  | CP-122 | Putative uncharacterized protein | Q3U8W9 | tr\|Q3U8W9\| |
|  | CP-160 | Protein S100-A6 | S10A6 | sp\|P14069\| |
|  | CP-187 | Ankyrin repeat domain-containing protein | ANKRD56 | sp\|Q8BZW2\| |
|  | CP-192 | Leucine-rich repeat-containing protein 59 | LRC59 | sp\|Q922Q8\| |
|  | CP-231 | Protein dpy-30 homolog | DPY30 | sp\|Q99LT0\| |
|  | CP-269 | Ryanodine receptor 1 | RYR1 | sp\|E9PZQ0\| |
